# A planar electronic acceptor motif contributing to NIR-II AIEgen with combined imaging and therapeutic applications†

**DOI:** 10.1039/d3sc06886b

**Published:** 2024-04-01

**Authors:** Ming Chen, Zhijun Zhang, Runfeng Lin, Junkai Liu, Meizhu Xie, Xiang He, Canze Zheng, Miaomiao Kang, Xue Li, Hai-Tao Feng, Jacky W. Y. Lam, Dong Wang, Ben Zhong Tang

**Affiliations:** a College of Chemistry and Materials Science, Jinan University Guangzhou 510632 China chenming@jnu.edu.cn; b Center for AIR Research, College of Materials and Engineering, Shenzhen University Shenzhen 518060 China wangd@szu.edu.cn; c Department of Chemistry, Hong Kong Branch of Chinese National Engineering Research Center for Tissue Restoration and Reconstruction, Guangdong-Hong Kong-Macau Joint Laboratory of Optoelectronic and Magnetic Materials, The Hong Kong University of Science and Technology Clear Water Bay, Kowloon Hong Kong 999077 China; d School of Science and Engineering, Shenzhen Institute of Aggregate Science and Technology, The Chinese University of Hong Kong Shenzhen (CUHK-SZ) Guangdong China tangbenz@cuhk.edu.cn; e AIE Research Center, Shaanxi Key Laboratory of Photochemistry, College of Chemistry and Chemical Engineering, Baoji University of Arts and Sciences Baoji 721013 China

## Abstract

Designing molecules with donor–acceptor–donor (D–A–D) architecture plays an important role in obtaining second near-infrared region (NIR-II, 1000–1700 nm) fluorescent dyes for biomedical applications; however, this always comes with a challenge due to very limited electronic acceptors. On the other hand, to endow NIR-II fluorescent dyes with combined therapeutic applications, trivial molecular design is indispensable. Herein, we propose a pyrazine-based planar electronic acceptor with a strong electron affinity, which can be used to develop NIR-II fluorescent dyes. By structurally attaching two classical triphenylamine electronic donors to it, a basic D–A–D module, namely Py-NIR, can be generated. The planarity of the electronic acceptor is crucial to induce a distinct NIR-II emission peaking at ∼1100 nm. The unique construction of the electronic acceptor can cause a twisted and flexible molecular conformation by the repulsive effect between the donors, which is essential to the aggregation-induced emission (AIE) property. The tuned intramolecular motions and twisted D–A pair brought by the electronic acceptor can lead to a remarkable photothermal conversion with an efficiency of 56.1% and induce a type I photosensitization with a favorable hydroxyl radical (OH˙) formation. Note that no additional measures are adopted in the molecular design, providing an ideal platform to realize NIR-II fluorescent probes with synergetic functions based on such an acceptor. Besides, the nanoparticles of Py-NIR can exhibit excellent NIR-II fluorescence imaging towards orthotopic 4T1 breast tumors in living mice with a high sensitivity and contrast. Combined with photothermal imaging and photoacoustic imaging caused by the thermal effect, the imaging-guided photoablation of tumors can be well performed. Our work has created a new opportunity to develop NIR-II fluorescent probes for accelerating biomedical applications.

## Abstract 2

We propose a pyrazine-based planar electronic acceptor with strong electron affinity which can be further utilized to design NIR-II fluorescence dyes with combined imaging and therapeutic applications.

## Graphical abstract

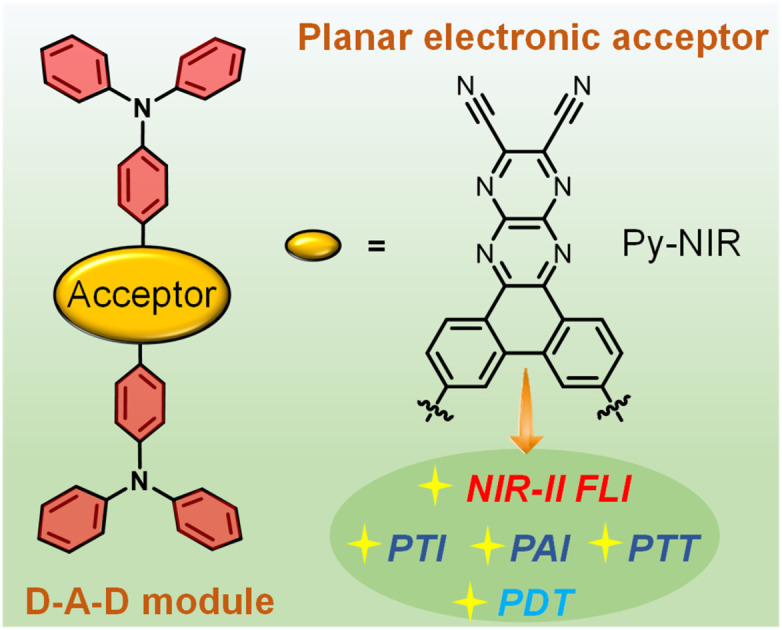

## Introduction

Fluorescence imaging is emerging as a promising tool in disease diagnosis for its noninvasiveness, real-time tracking, high resolution and excellent sensitivity.^1^ However, compared to traditional imaging techniques such as magnetic resonance imaging, positron emission tomography, ultrasonic imaging, *etc.*, the investigation of fluorescence imaging in clinical application is still in its infancy stage as the bottleneck of low penetrability in biological tissue is always encountered.^2^ This issue can be alleviated by using fluorescent dyes with second near-infrared region (NIR-II, 1000–1700 nm) emission as imaging agents which can dramatically promote imaging depth and resolution by reducing light scattering and absorption from the biological tissue as well as biological autofluorescence.^3^ Up to now, much effort has been devoted to develop NIR-II fluorescent dyes such as single-walled carbon nanotubes, quantum dots, up-conversion nanoparticles, organic dyes and so on.^4^ Among these fluorescent materials, organic fluorogens have appeared to be the most attractive candidates for NIR-II imaging due to their good biosafety and conceivable properties based on flexible molecular design. Early investigation has focused on polymethine/cyanine dyes with planar, extended and rigid conjugated structures.^5^ Although most of these dyes are well studied and commercially available, the drawbacks of strong self-absorption caused by a small Stokes shift, aggregation-caused fluorescence quenching in the aqueous environment, and poor stability brought out by the photo-oxidation of inner double bonds have hampered their practical applications.^6^

**Fig. 1:**
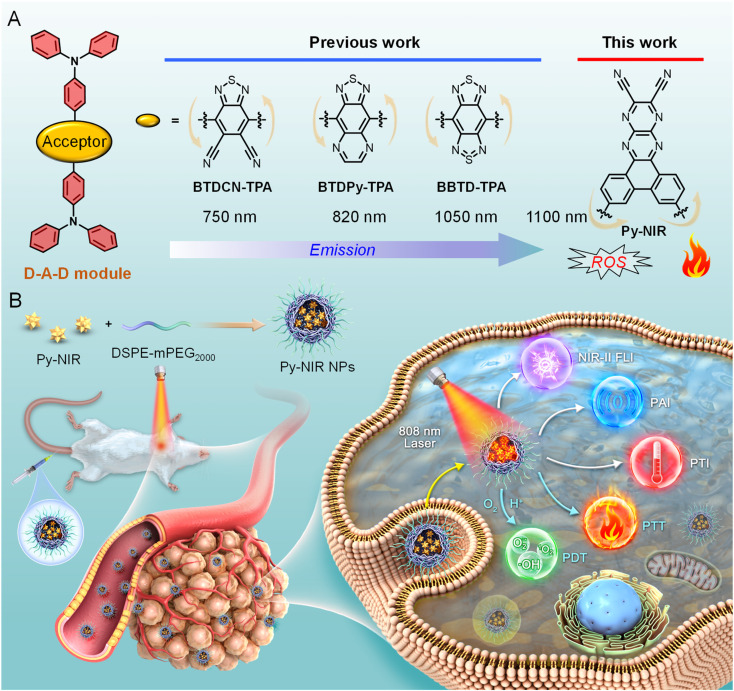
(A) The structure evolution of BTD-related electronic acceptors to generate NIR-II fluorescent dyes and the proposed pyrazine-based planar electronic acceptor in this work. (B) Graphical representation of combined imaging and therapeutic applications of Py-NIR NPs towards orthotopic 4T1 breast tumor bearing mice.

Aggregation-induced emission luminogens (AIEgens) are a class of organic dyes with twisted and flexible conformation.^7^ This factor enables them to possess a large Stokes shift due to vigorous structural relaxation between the ground and excited states and high luminescence efficiency in the aggregate state by the restriction of excited-state intramolecular motion. The aggregation also brings a good stability and resistance to photo-bleaching. All these bestow them with excellent imaging and therapeutic applications.^8^ To expand AIEgens in NIR-II imaging, the design of a molecule with donor–acceptor–donor (D–A–D) architecture is favorable using the combination of good mobility and strong intramolecular charge transfer between the electronic donors and acceptor.^9^ However, while the structure of the donor is versatile and devisable, choosing the acceptor is much important but difficult because it plays a major role in the electronic structure of the dye molecule and most of the acceptors show a poor electron affinity. The past decade has witnessed the development of advanced acceptors to contribute to NIR-II dyes. It reveals that after structural derivation from a typical electronic acceptor of benzothiadiazole (BTD), an extremely strong acceptor unit named benzobisthiadiazole (BBTD) is successfully generated which has occupied a dominant role in designing NIR-II dyes with a D–A–D structure (Fig. 1A).^10^ For example, in 2016, Dai and his co-workers synthesized a BBTD-based D–A–D dye CH1055 with its emission maximum at 1055 nm for imaging mouse blood and lymphatic vasculatures, tumors and lymph node mapping.^11^ Subsequently, plenty of studies have been stimulated to boost *in vivo* fluorescence imaging applications by tuning the emission wavelength and efficiency of such AIE dyes through molecularly engineering the shielding, electronic and volumetric effects of the donors.^12^ While BBTD prevails in NIR-II AIEgen design, the development of other electronic acceptor units is very limited. Therefore, developing new acceptor structures can not only enrich NIR-II AIEgens but also provide more properties for accelerating practical biomedical applications.

Organic fluorogens with phototherapeutic properties such as photothermal therapy (PTT) and photodynamic therapy (PDT) have drawn much attention in practical applications due to their good curative effect, low drug resistance, fewer side effects, *etc.*^13^ PTT and PDT have compensatory properties in treatment and uniting them together may result in a synergistic curative effect with “1 + 1 > 2”.^14^ For example, as the tumors are anaerobic, the incorporation of the photothermal effect would improve the oxygen supply in the tumor tissue by accelerating the blood flow, which is beneficial to produce reactive oxygen species (ROS) to enhance the PDT effect and kill the heat-resistant tumor cells in PTT. However, according to the energy conservation rule, these processes, together with fluorescence emission, are always competitive after molecular excitation, and it is challenging to balance these properties to maximize the combined imaging and therapeutic effect. To realize effective photothermal conversion, tailoring the molecules with flexible conformation is essential because it may facilitate the intramolecular motions for heat dissipation in the excited state.^15^ On the other hand, the ROS is produced from the excited triplet through intersystem crossing (ISC) from the excited singlet by the energy transfer or photo-induced electron transfer process.^16^ To enhance the ISC, a small singlet-triplet splitting (Δ*E*_ST_) is required especially in a twisted D–A molecular system where the electronic clouds have a negligible overlap between the ground and excited states.^17^ However, these properties need elaborate molecular design by introduction of intramolecular rotators or reducing intermolecular interactions to promote intramolecular motions and providing an additional steric effect to twist the D–A pair.^18^ The search for simple D–A–D molecular module to concurrently and directly obtain balanced NIR-II fluorescence imaging as well as phototherapeutic properties is particularly desirable.

In this work, we develop a pyrazine-based planar electronic acceptor to design NIR-II fluorescent dyes with combined imaging and therapeutic applications (Fig. 1). We propose a basic D–A–D module by structurally attaching two typical triphenylamine electronic donors to such an acceptor, and a dye named Py-NIR is generated with its properties affected by the acceptor well discussed. Py-NIR shows good processability and can be fabricated into water-dispersed nanoparticles (NPs) capable of excellent stability and biosafety. Due to the extremely strong electron affinity of the planar acceptor, remarkable intramolecular charge transfer can take place between the donor and acceptor to induce an obvious NIR-II fluorescence emission at ∼1100 nm in the Py-NIR NPs. Meanwhile, the good planarity of the electronic acceptor plays a positive role in increasing the irradiative transition while decreasing the non-radiative transition, which is favorable for luminescence. The AIE effect of Py-NIR is caused by the flexible molecular conformation provided by the specific construction of the acceptor to increase the repulsion between the connected donors. Although the excited-state intramolecular motions have been reduced by the introduction of the planar electronic acceptor, it is still drastic to induce a high photothermal conversion efficiency of 56.1%. Moreover, the twisted D-A pair caused by the acceptor in Py-NIR enables it to possess a small Δ*E*_ST_ of 0.14 eV, which is beneficial for the ISC to enhance triplet sensitization, while such an acceptor can also induce an evident hydroxyl radical (OH˙) formation through a type I mechanism. Therefore, with the aid of the planar electronic acceptor, congenerous NIR-II fluorescence, photothermal conversion and photosensitization can be achieved readily based on a sample D–A–D module of Py-NIR. The intracellular ROS generation as well as *in vitro* phototoxicity of Py-NIR NPs are investigated based on mouse breast cancer 4T1 cells before the *in vivo* experiment. Moreover, by employing the Py-NIR NPs, the NIR-II fluorescence imaging of orthotopic 4T1 breast tumors in mice can be collected with a good target ability, high sensitivity and contrast. By taking advantage of the good photothermal properties of Py-NIR NPs, photothermal imaging (PTI) and photoacoustic imaging (PAI) can be conducted which may have compensatory properties for fluorescence imaging. In addition, guided by multiple imaging models, the synergetic phototherapy from Py-NIR NPs can cause a dramatic ablation of tumors with the curative mechanism well studied by the histological and immunohistochemical analyses. The planar electronic acceptor in this work will provide a motif to spark more NIR-II fluorescent dyes with combined functions to boost biomedical applications.

## Results and discussion

Previous studies reveal that after structural decoration of BTD from nitrile groups to heterocycles on another side of phenyl groups, the electron affinity of resulting units can be greatly improved, and it is well accepted that BBTD has become a powerful acceptor to construct D–A–D dyes with NIR-II emission.^19^ Our group has been engaged in the investigation of fluorescent dyes based on pyrazine building blocks.^20^ We find that a pyrazine-based planar electronic acceptor is devisable by a cyclization reaction of 5,6-diaminopyrazine-2,3-dicarbonitrile with 9,10-phenanthrenequinone, which has potential in the design of NIR-II fluorescent dyes due to the extremely strong electron affinity (Fig. 1A). To achieve such a target, a D–A–D luminogen, namely Py-NIR, is designed by structurally attaching two typical triphenylamine donors onto the acceptor core, and it reflects a basic D–A–D module to reveal the properties of such kinds of NIR-II dyes. The design concept is shown below: (1) the planarity of the electronic acceptor can not only improve the electron-withdrawing ability to induce a remarkable intramolecular charge transfer from donors to acceptors to generate NIR-II fluorescence but also pose limitations to intramolecular motions to benefit the emission, (2) unlike BBTD-based D–A–D dyes, the “V”-type connection of the donors and acceptor can cause an obvious repulsion between donors due to the steric effect to produce twisted and flexible molecular conformation, which is contributive to the combined AIE effect and photothermal conversion in the aggregate state, and (3) the distortion between the donor and acceptor can lead to an obvious orbital separation in the space between the ground and excited state which is crucial to reduce the Δ*E*_ST_ to enhance the ISC for triplet sensitization. All these features may endow Py-NIR with multiple functions which is very promising in synergetic biomedical applications.

**Fig. 2:**
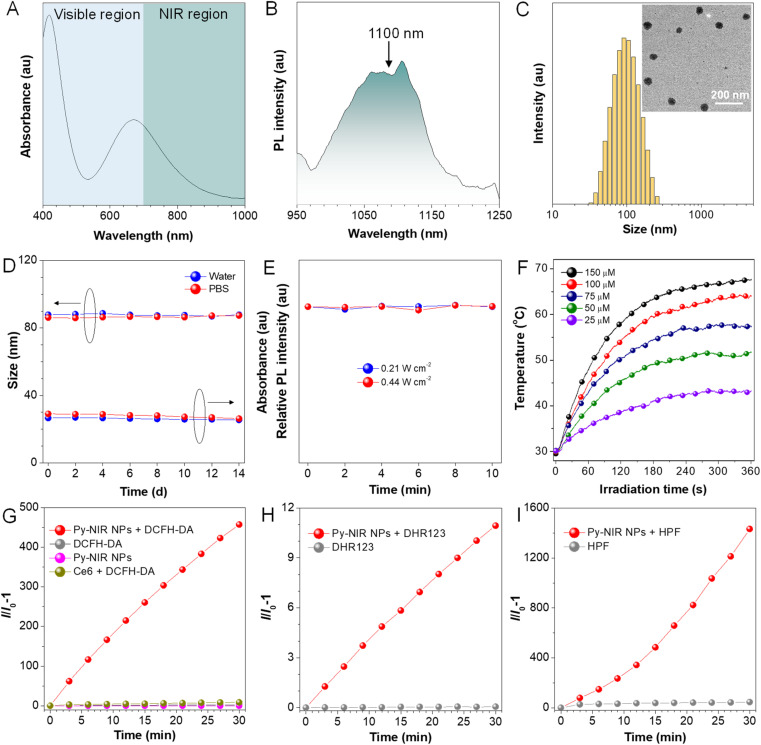
(A) UV-Vis and (B) PL spectra of Py-NIR NPs in water. (C) DLS of Py-NIR NPs. Inset: their TEM image. (D) Changes in the particle diameter and absorption intensity of Py-NIR NPs during two-week storage. (E) PL intensity of Py-NIR NPs under continuous 10-minute laser irradiation. (F) Photothermal curves of Py-NIR NPs with different concentrations under consistent laser irradiation (1.0 W cm^−2^). Changes in relative emission intensities of (G) DCFH-DA, (H) DHR 123 and (I) HPF in the presence of Py-NIR NPs under continuous 808 nm laser irradiation (1.0 W cm^−2^).

Py-PS is synthesized facilely in a two-step reaction (Scheme S1†). An intermediate of triphenylamine-substituted 9,10-phenanthrenequinone (3) is first synthesized between 3,6-dibromo-9,10-phenanthrenequinone (1) and 4-(diphenylamino)phenylboronic acid (2) by a classical Suzuki coupling reaction. Then, the reflux of 3 and 5,6-diaminopyrazine-2,3-dicarbonitrile (4) in 1,2,4-trimethylbenzene in the presence of *p*-toluenesulfonic acid as a dehydrant can afford a black cyclized product of Py-NIR after purification by column chromatography. Py-NIR was characterized by ^1^H and ^13^C NMR and high-resolution mass spectroscopies with satisfactory data corresponding to its structure (Fig. S1–S5†). The thermogravimetric analysis (TGA) indicates that its temperature for 5% weight loss is higher than 420 °C, suggestive of a good thermal stability (Fig. S6†). Like most fluorogens, Py-NIR is hydrophobic due to its aromatic structure. However, its good solubility in common organic solvents such as tetrahydrofuran and chloroform endows it with a good processability. Then, the water-dispersed nanoparticles of Py-NIR (Py-NIR NPs) are prepared through a nanoprecipitation method with 1,2-distearoyl-*sn*-glycero-3-phosphoethanolamine-*N*-[methoxy(polyethylene glycol)-2000] (DSPE-mPEG_2000_) as a matrix, which is suitable for further property and application investigations. The encapsulation efficiency of Py-NIR NPs is deduced to be 91.2%. The UV-Vis spectrum shows that there are two absorption peaks at 420 nm and 670 nm for Py-NIR NPs in the range from 400–1000 nm (Fig. 2A), which are mainly attributable to the local state transition and intramolecular charge transfer from the donors to the acceptor, respectively. Besides, the photoluminescence (PL) investigation suggests that a distinct emission peaking at ∼1100 nm can be recorded with the NPs in the presence of 660 nm laser excitation, while only a weak signal is found when it is molecularly dissolved in the solution under the same excitation conditions (Fig. 2B and S7†). Thus, Py-NIR is a typical AIEgen with NIR-II fluorescence characteristics. The emission maximum of Py-NIR NPs is ∼50 nm longer than that of the BBTD-based D–A–D dye (BBTD-TPA) containing triphenylamine donors with a similar construction.^10*c*^ The PL quantum efficiency of Py-NIR NPs is deduced to be 0.07% by a relative method with indocyanine green (ICG) as a standard (Fig. S8†).^21^ Note that the strong electronic acceptor has contributed greatly to the NIR-I absorption and NIR-II emission, while such a property is particularly useful to improve the imaging depth and resolution by reducing light scattering and absorption from biological tissue and biological self-fluorescence interference. Moreover, the Stokes shift of Py-NIR NPs is recorded as ∼430 nm, which is much larger than that of BBTD-TPA (350 nm).^10*c*^ The large Stokes shift suggests that undesirable self-absorption in practical application can be effectively avoided.

Dynamic light scattering (DLS) reveals that the Py-NIR NPs possess an average diameter of 87 nm in the water, while the spherical particles with an almost uniform size of ∼60 nm can be observed in the transmission electron microscope (TEM) image (Fig. 2C). The smaller size obtained by TEM than DLS is due to the shrinkage of NPs in the ultra-dry state under high vacuum during the TEM test.^22^ Then, the stability of Py-NIR NPs is evaluated. As monitored by DLS in the continuous two weeks, there are fewer changes in size of NPs both in the water and phosphate-buffered saline (PBS), suggestive of a good size stability in storage (Fig. 2D). Moreover, during the same period, the UV-Vis spectra exhibit an ignorable change both in the absorption profile and intensity in water and PBS, implying that they have a good colloidal stability without precipitate formation (Fig. 2D and S9†). The photostability of fluorescent dyes is important since it may affect imaging resolution and durability. To check it, the PL spectra of Py-NIR NPs are recorded under continuous 10-minute 660 nm laser irradiation. It is clear that there is almost no loss in the fluorescence intensity under persistent laser irradiation with different powers, proving that the Py-NIR NPs possesses an excellent resistance to photobleaching (Fig. 2E).

We then examine whether good photothermal conversion properties could be realized based on our design principle. The photothermal capacity of Py-NIR NPs is measured under 808 nm laser irradiation (1.0 W cm^−2^) for 6 minutes. It can be observed that the temperature of Py-NIR NPs increases sharply in the first 4 minutes as the time elapses and almost reaches a constant afterwards in a concentration range of 25–150 μM (Fig. 2F). Increasing the NP concentration can lead to a faster temperature elevation and the maximum temperature increment is recorded to be ∼38 °C with a concentration of 150 μM. The photothermal conversion effect is positively correlated with the laser power, and adopting a suitable power can raise the temperature above 70 °C (Fig. S10A†). The photothermal conversion efficiency of Py-NIR NPs is evaluated to be 56.1% by the reported method,^23^ which is superior to that of the previously reported Au nanorods (28.5%), inorganic quantum dots (31.6%), semiconducting polymers (30.0%), BODIPY derivatives (23.0%) and so forth.^24^ Moreover, the photothermal stability of Py-NIR NPs is investigated with a repeating heating-cooling experiment, and it suggests that there is no decline in the photothermal behavior even after six cycles (Fig. S10B†). The good photothermal conversion effect, along with the excellent stability, exhibits great potential for further application with the NPs.

The photosensitization properties of Py-NIR NPs are also studied with the aid of different indicators such as dichlorofluorescein diacetate (DCFH-DA), 9,10-dimethylanthracene (ABDA), dihydrorhodamine (DHR) 123, and hydroxyphenyl fluorescein (HPF), which are normally used to evaluate the overall ROS, singlet oxygen (^1^O_2_), superoxide radical (O_2_˙^−^) and hydroxyl radical (OH˙), respectively. The PL spectra of DCFH-DA in coexistence with the NPs under 808 nm laser irradiation (1.0 W cm^−2^) display a persistent fluorescence enhancement as time increases (Fig. S11†). At 30 min, the fluorescence signal has been strengthened by more than 4 × 10^2^-fold, while fewer changes can be detectable only in the presence of DCFH-DA or NPs upon the same laser irradiation (Fig. 2G). However, no fluorescence signal is observable based on a commercial photosensitizer (chlorin e6, Ce6) under the same excitation conditions mostly because of the absence of absorption in the NIR region. Thus, the ROS can be generated efficiently with our dye by NIR light excitation. Further investigation indicates that the UV-Vis spectra of ABDA have dropped somewhat with the irradiation time, and almost 6% of the intensity is lost at 30 min, suggestive of a weak photosensitization effect to generate ^1^O_2_ (Fig. S12†). At the same time, the production of O_2_˙^−^ is also faint as reflected by only a ∼10-fold fluorescence enhancement of DHR 123 under the light excitation (Fig. 2H and S13†). By contrast, the fluorescence peak of HPF has increased remarkably by more than 1.4 ×10^3^-fold in the presence of NPs and a laser, proving that an efficient generation of OH˙ has been achieved (Fig. 2I and S14†). The above results suggest that the photosensitization properties of Py-NIR NPs are mainly managed by the type I process, which is much desirable in the treatment of anaerobic tumors due to its lower dependence on oxygen. Moreover, Py-NIR NPs have a much faster rate to generate OH˙ than other ROS. Since OH˙ has been regarded as one of the most toxic ROS as it can react with most biological substrates at the site where it is produced, such a photosensitization effect is extremely favorable in practical PDT application.

**Fig. 3:**
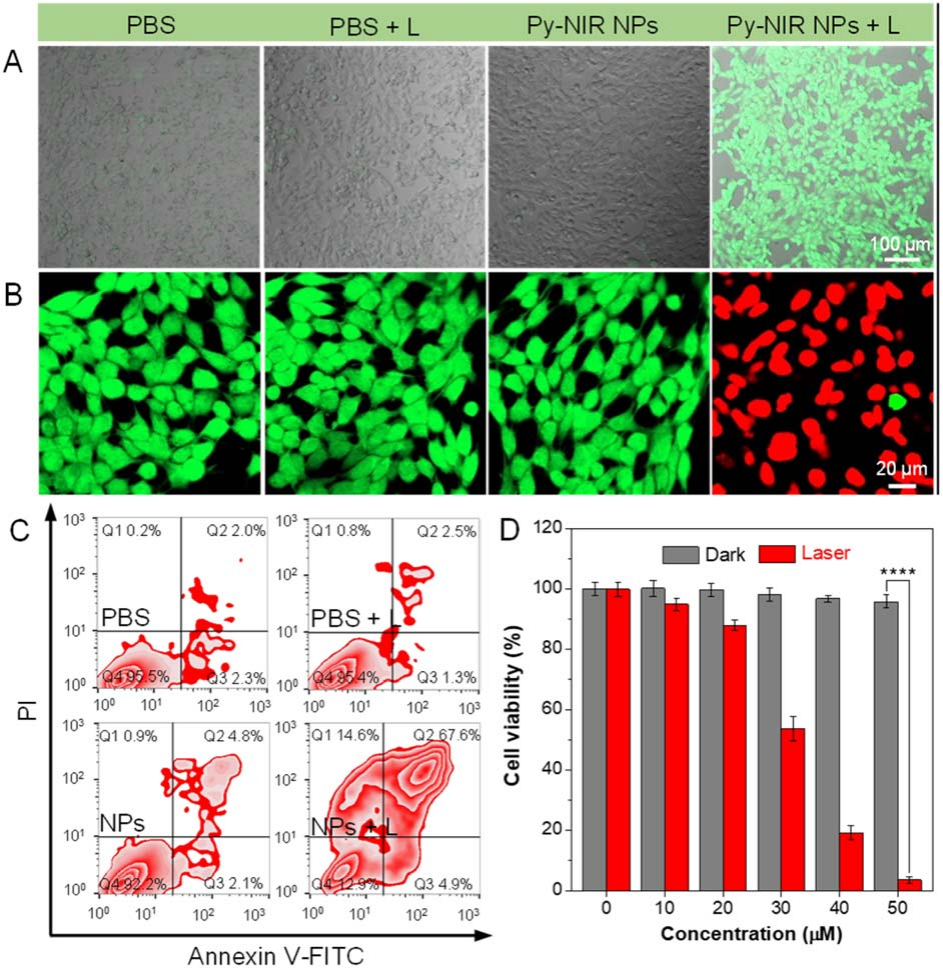
CLSM imaging of 4T1 cells for (A) detecting intracellular ROS and (B) live/dead cell staining with treatment of PBS, PBS + L, Py-NIR NPs, and Py-NIR NPs + L, respectively. Evaluation of (C) apoptosis and (D) cell viability of 4T1 cells with different treatments (mean ± SD, *n* = 6), ****p < 0.0001. Student's *t*-test.

We also evaluate the photosensitization behavior of Py-NIR NPs at the cellular level. To conduct the experiment, four groups of mouse breast cancer 4T1 cells are adopted, which are treated with PBS, PBS + laser (L), Py-NIR NPs and Py-NIR NPs + L, respectively. DCFH-DA is used as an intracellular ROS indicator, because if the ROS are produced, it would be oxidized to form a product with detectable green fluorescence. The laser scanning microscopy (CLSM) results indicate that only the cells stained with the NPs and irradiated using the 808 nm laser (1.0 W cm^−2^) can generate a distinct green fluorescence signal (Fig. 3A). In contrast, almost no fluorescence can be observed in the control groups. This suggests that the ROS has been generated vigorously from the NPs in the cells by the light excitation, which agrees well with the above spectral analysis. Then, their phototoxicity towards the 4T1 cells is investigated using fluorescein diacetate/propidium iodide (FDA/PI) as an indicator to distinguish the live/death cells. The results show that only strong green fluorescence from FDA can be collected in the groups of PBS, PBS + L and Py-NIR NPs, proving that the cells are intact under these conditions since no ROS or heat can destroy them (Fig. 3B). In sharp contrast, in the Py-NIR NPs + L group, these collective factors may be generated from the NPs which is vital to induce apoptosis, while almost the red fluorescence from PI can be recorded. The apoptosis of cells by the collaborative PDT-PTT effect is further explored based on Annexin V-FITC/PI assay as reflected by a high apoptotic ratio of ∼87.1% (Fig. 3C). Besides, the CCK-8 assay indicates that the cells are almost safe within a NP concentration from 0–50 μM in the dark. The good cell compatibility of Py-NIR NPs is also proved by co-culturing with normal cells with an ignored toxicity (Fig. S15†). However, after light irradiation, an increased phototoxicity can be observable as the NP concentration increases (Fig. 3D). And at a concentration of 50 μM, more than 95% of the cells have been killed by the remarkable phototherapeutic effect.

**Fig. 4:**
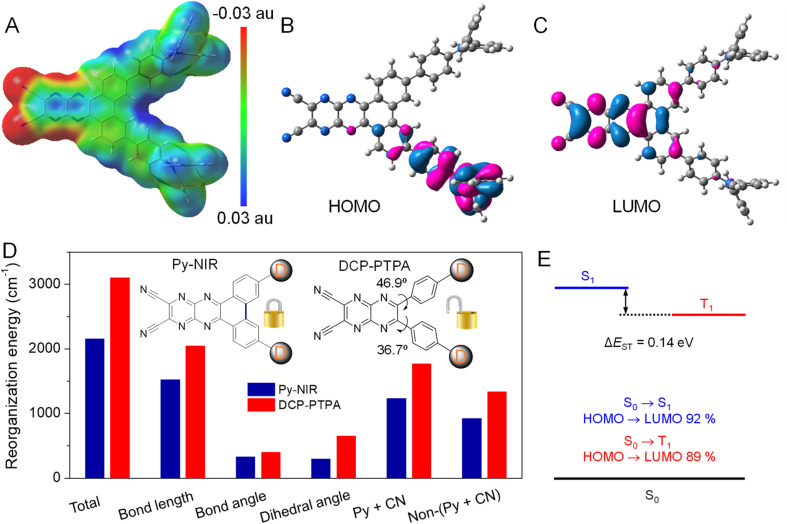
(A) Electrostatic potential surface based on optimized S_1_ geometry of Py-NIR. (B) HOMO and (C) LUMO distributions on S_1_ geometry. (D) Total *λ* and the *λ* of bond length, bond angle, dihedral angle, pyrazine and nitrile moieties (Py + CN), and non-pyrazine and nitrile moieties (Non-(Py + CN)) in Py-NIR and DCP-PTPA. (E) Energy diagram based on S_1_ geometry, energy levels of S_1_ and T_1_, and electronic configuration of S_1_ and T_1_.

Since the obvious NIR-II fluorescence, good photothermal conversion and photosensitization effects have been achieved in Py-NIR, how does the introduction of a planar electron acceptor into the D–A–D structure affect these properties? To unveil this puzzle, theoretical simulation based on Py-NIR is carried out. To better clarify the multiple properties brought by the planar effect of the electronic acceptor, a nonplanar acceptor with a similar structure while the inner phenyls are unlocked, is taken as a control. Fortunately, a D–A–D dye named DCP-PTPA has been synthesized by substituting such an acceptor with the same donors in our previous work.^25^ The optimized S_1_ confirmations indicate that both Py-NIR and DCP-PTPA are highly twisted as the triphenylamine branches repel each other by the strong steric effect, which is inherently determined by the unique “V”-type connection mode contributed by the acceptors. From the molecular electrostatic potential maps, the most negative electrostatic potential surfaces which are represented by the red are dominated by the N atoms from the nitrile groups and pyrazine rings in the acceptors, which is decided from the strong electronegativity of N atoms (Fig. 4A and S16A†). In contrast, the positive electrostatic potential surfaces marked with blue are mainly located in the triphenylamine moieties due to the electron-donating effect shared by the long pair electrons from the N atoms with the phenyl rings. The remarkably opposite electronic effects of acceptors and donors may induce a dramatic intramolecular charge transfer between them under excitation to red-shift the emissions. This is verified by the results that their highest occupied molecular orbitals (HOMOs) and lowest unoccupied molecular orbitals (LUMOs) separately occupy the donors and acceptors with a very small electron cloud overlapping (Fig. 4B, C, S16B and C†). However, employing a planar electronic acceptor is much better to improve the electronic affinity to enhance the charge transfer in a D–A–D system to obtain a NIR-II emission. This matches well with the fact that the emission wavelength of Py-NIR is recorded to be ∼1100 nm by PL analysis, which is ∼200 nm longer than that of DCP-PTPA.^25^

Moreover, the oscillator strengths of Py-NIR and DCP-TPA are calculated to be 0.16 and 0.14, respectively, suggesting the acceptor planarity plays a positive role in enhancing radiative transition. On the other hand, the reorganization energy (*λ*) of these molecules is investigated using a MOMAP package,^26^ as it is one of the key factors in evaluating the role of intramolecular motions on the nonradiative decay process. The total *λ* for Py-NIR and DCP-TPA are calculated to be 2154 and 3101 cm^−1^, respectively (Fig. 4D). This suggests that, in comparison to DCP-PTPA, the nonradiative transition of Py-NIR caused by the intramolecular motions has been obviously suppressed due to the introduction of the planar electronic acceptor. To get a deeper insight into the structure–property relationship, the *λ* contributed by changes in bond length, bond angle, and dihedral angle in each molecule are further evaluated. The results indicate that the motions of bond stretching and group rotation dominate the *λ* of Py-NIR and DCP-PTPA by their large proportions, while both *λ* in the former are much smaller than those in the latter. This suggests that these motion models play a major role in excited-state conformational relaxation, and locking the phenyls in the acceptor can cause a limitation to such motions. Since the bond length variation mainly comes from the bond stretching vibration in the pyrazines and nitrile groups, the *λ* of this part has reduced as the locking of phenyl rings can enhance the planarity of the acceptor to benefit electron communication with them to homogenize the charge distribution, while the bond length variation caused by the charge redistribution in the pyrazine acceptor between the ground and excited states is decreased (Fig. 4D). Meanwhile, the phenyl locking can obviously remove the group rotation met by the acceptor in DCP-PTPA to reduce the change in the dihedral angle, which can be proved by the decreased *λ* in the phenylamine branches (non-pyrazine and nitrile regions). However, the additional phenyl rotation in the triphenylamine branches is favorable to the AIE effect, while such motions are easy to take place in the solution but suppressed in the aggregate state. Thus, the enhanced radiative decay as well as the limited nonradiative decay brought by the planar electronic acceptor collectively boost the NIR-II fluorescence emission. Moreover, as the intramolecular motions can accelerate heat dissipation of the excited state after excitation, such balanced excited-state properties in Py-NIR can still result in a rapid photothermal conversion with a high efficiency of 56.1%.

The “V”-type construction of Py-NIR also endows it with a small Δ*E*_ST_ of 0.14 eV as deduced by the calculation because the electron exchange energy decreases with the spatial separation of the HOMO–LUMO wave functions (Fig. 4E).^27^ Moreover, large transition proportions have been found in the electronic configuration of S_1_ and T_1_, suggesting a high probability for the ISC from S_1_ to T_1_. These factors may enable an effective ISC process from S_1_ to T_1_, where the sensitization effect can be produced. In our previous investigation, we found that the photosensitization effect is closely related to intramolecular motions and this suggests that the more intramolecular motions in the photosensitizer, the much stronger the type I photosensitization with OH˙ generation.^28^ Our study reveals that the production of OH˙ from Py-NIR is much stronger than that of ^1^O_2_ and O_2_˙^−^. However, under the same conditions, the formation rate of ^1^O_2_ in DCP-PTPA is slower than that of Py-NIR, while that of O_2_˙^−^ and OH˙ shows an enhancement effect.^28^ Because intramolecular motions have been reduced in Py-NIR by the introduction of the planar electronic acceptor, the type I process to generate O_2_˙^−^ and OH˙ is suppressed as the photo-induced electron transfer may require a transient molecular collision with O_2_ in the excited state.^29^ As compensation, many opportunities can be obtained in the excited state to participate in the type II process to form ^1^O_2_, which always involves a Dexter energy transfer with an electron cloud overlapping between the host and guest.^30^ Therefore, our results are well consistent with the viewpoint proposed before and the photosensitization behavior of Py-NIR can be finely tuned using the structural factor.

**Fig. 5:**
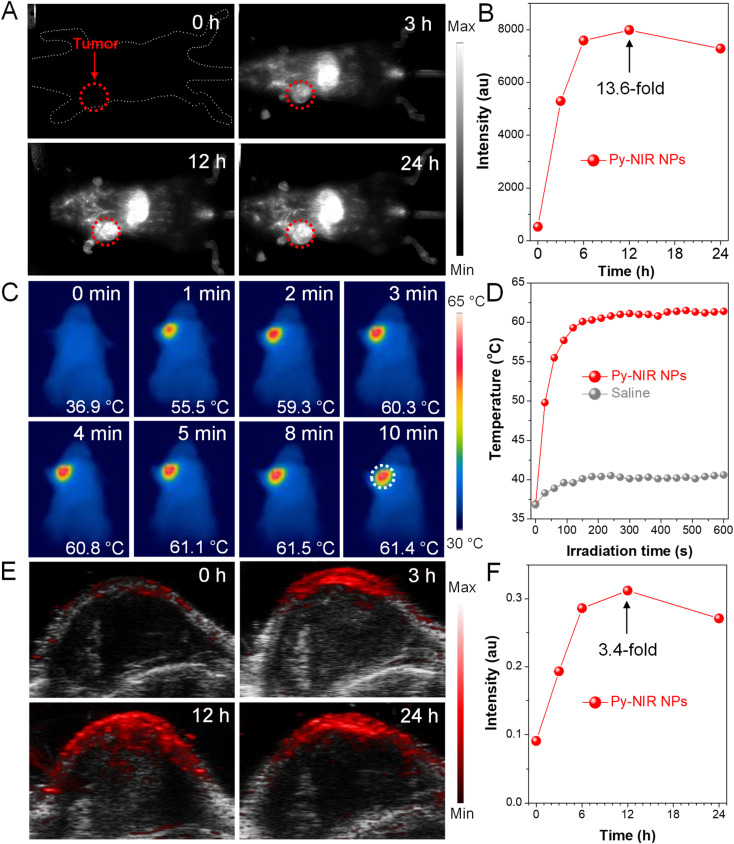
(A) NIR-II fluorescence imaging of 4T1 breast tumor bearing mice with Py-NIR NPs at different injection times. (B) Plot of fluorescence intensity *versus* injection time. (C) PTI of 4T1 breast tumor bearing mice at 12 h post-injection of the Py-NIR NPs under consistent 10-minute laser irradiation (1.0 W cm^−2^). (D) Plot of temperature in mice injected with Py-NIR NPs and saline *versus* irradiation time. (E) PAI of 4T1 breast tumors at different post-injection times of Py-NIR NPs under laser excitation (1.0 W cm^−2^). (F) Plot of PAI intensity *versus* injection time.

Encouraged by the excellent properties of Py-NIR NPs, we evaluate their utility as NIR-II probes for *in vivo* imaging applications based on an orthotopic 4T1 breast tumor bearing mouse model. We first investigate whether the NIR-II fluorescence provided by the probe could image the tumors. The results show that there is almost no fluorescence signal in the NIR-II window which can be detected at the tumor site before the administration of Py-NIR NPs, suggestive of low biological autofluorescence interference of NIR-II fluorescence imaging (Fig. 5A). However, after injection of the NPs *via* the tail vein, the NIR-II fluorescence signal increases rapidly as time passes, which is due to the increasing accumulation of NPs into tumors caused by the enhanced and permeability retention (EPR) effect. The boundary of tumors becomes clear owing to the superior sensitivity of fluorescence imaging. The fluorescence has reached a maximum at 12 h post-injection of the NPs, which is 13.6-fold higher than that before the injection, suggesting a good imaging contrast (Fig. 5B). After that, the fluorescence signal decreases gradually as a result of the metabolism of the NPs through the circulatory system. To further confirm the anchoring of NPs into the tumors, the excised NIR-II fluorescence imaging experiment is carried out. The mice are sacrificed at 24 h post-injection, while the main organs such as the heart, liver, spleen, lung and kidney, and tumor are extracted. It is clear that the bright fluorescence signal can be found in the tumor, manifesting a good target capacity of the NPs towards it (Fig. S17†). Moreover, obvious imaging signals have also appeared in the liver and spleen, while negligible signals can be observed in the other organs. The high accumulation of Py-NIR NPs in the liver and spleen should be attributed to the fact that both of them are the major organs of the mononuclear phagocytic system (MPS). The above results show that the Py-NIR NPs are competent for cancer diagnosis with good target ability, high sensitivity and contrast.

Thanks to a strong photothermal conversion of Py-NIR NPs, their *in vivo* tumor PTI and PAI are also performed due to a direct or indirect heat effect, which may possess compensatory properties for fluorescence imaging. The photothermal images of the tumor site after 12 h post-injection of Py-NIR NPs exhibit an identical temperature to the body without the 808 nm laser irradiation (Fig. 5C). After irradiation for 2 min (1.0 W cm^−2^), the temperature of the tumor has increased sharply from 36.9 °C to 59.3 °C (Δ*T* = 22.4 °C) and almost stays constant afterwards (Fig. 5D). As a control, only a weak temperature increase of ∼3 °C can be found in the mice treated with saline under the same excitation conditions (Fig. 5D and S18†). The dramatic temperature elevation brought by the Py-NIR NPs can not only provide an additional imaging modality but also cause thermal damage to the cancer cells in the treatment. Moreover, we also study the PAI performance of Py-NIR NPs towards the *in vivo* tumors using an 808 nm laser (1.0 W cm^−2^). It suggests that there is a weak PA signal in the tumor site before the NP injection, which is due to the absorption from background substrates such as endogenous melanin and hemoglobin (Fig. 5E). The PA intensity improves obviously with time after the NP administration and shows a maximum enhancement of 3.4-fold at 12 h (Fig. 5F). The change in PA intensity shows a very similar profile to that of NIR-II fluorescence imaging, which is affected by the concentration of accumulated NPs. Note that although PAI always usually possesses a high spatial resolution in the *in vivo* imaging, its sensitivity and contrast are still worse than fluorescence imaging as revealed by a weaker signal presentation and stronger background at the tumor sites.

**Fig. 6:**
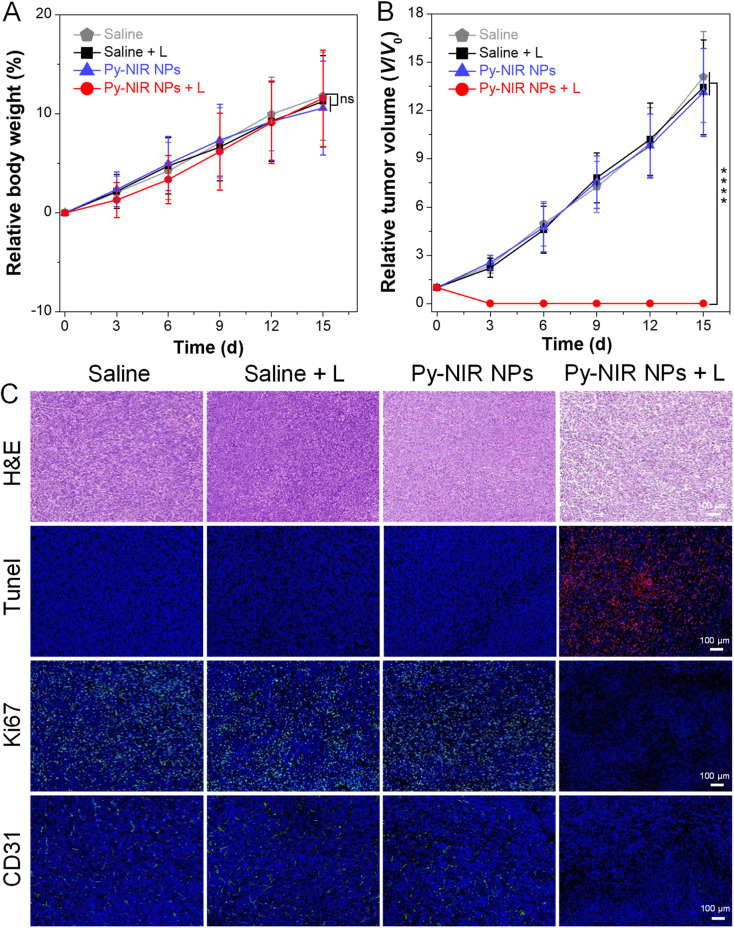
(A) Changes in body weight of mice with treatment of saline, saline + L, Py-NIR NPs, and Py-NIR NPs + L, respectively during the 15-day study duration (mean ± SD, *n* = 6), ns: not significant. Student's *t*-test. (B) Changes in tumor volume with different treatments during the 15-day study duration (mean ± SD, *n* = 5), ****p < 0.0001. Student's *t*-test. (C) H&E, Tunel, Ki67 and CD31 staining of tumor tissues on day 15 with different treatments.

The combined imaging modes, together with good photothermal and photosensitization properties enable Py-NIR NPs to emerge as a useful tool in the imaging-guided phototherapy of tumors by NIR light excitation. To further evaluate the antitumor effect, the orthotopic 4T1 breast tumor bearing mice with tumor size reaching ∼100 mm^3^ are randomly divided into four groups (*n* = 5 for each group) with different treatments, which are saline, saline + L, Py-NIR NPs, and Py-NIR NPs + L, respectively. If necessary, the 808 nm laser (1.0 W cm^−2^) is employed to irradiate the tumor region for 10 min at 12 h after intravenous injection of the saline or NPs. For all the groups, the body weight and tumor volume of mice are recorded every 3 days over a period of 15 days. It is clear that there is almost no change in the mouse body weight in the four groups during the same study duration, suggesting that their growth behavior is less affected by these treatments (Fig. 6A). However, the tumor volume in the Py-NIR NPs + L group has been obviously suppressed on day 3 while a negligible size can be observed in the extending time (Fig. 6B and S19†). In sharp contrast, the tumor volumes in the control groups increase gradually with time and almost reach ∼13-fold on day 15. These suggest that the Py-NIR NPs have a superior *in vivo* phototherapeutic effect to ablate the tumor under NIR light excitation, which is in good accordance with the observation in the *in vitro* phototoxicity experiment. Moreover, the therapeutic mechanism in terms of cancer cell death is evaluated by histological and immunohistochemical analyses of the extracted tumor tissues (Fig. 6C). The hematoxylin and eosin (H&E) staining result indicates that there is significant cancer cell damage brought by the phototherapy of NPs, while the cells in saline, Py-NIR NPs, and saline + L groups retain their normal morphology. The terminal deoxynucleotidyl transferase dUTP nick end labeling (Tunel) immune-fluorescence staining shows that a dramatically increased population of apoptotic cells reflected by the red fluorescence can only be found in the group with the phototherapy. The expression of the proliferative marker (Ki67) in the Py-NIR NPs + L group is much lower than that in the others, suggesting that the tumor cell proliferation and growth have been remarkably prohibited under the therapeutic conditions. Moreover, as the endothelial cancer marker, platelet endothelial cell adhesion molecule-1 (CD31) displays distinct green fluorescence in saline, Py-NIR NPs, and saline + L groups, while no such signal is found in the Py-NIR NPs + L group. This suggests that the phototherapeutic effect caused by the NPs could suppress tumor angiogenesis, which may cut off the oxygen and nutrient supply for tumor growth. Collectively, the synergetic imaging and therapeutic outcomes exhibit great promise in the clinical application.

Finally, the biosafety of Py-NIR NPs is assessed as it plays an indispensable role in the practical application. First, the biochemistry and blood routine indices are studied by analyzing the blood samples extracted from the mice on day 15. The blood biochemistry parameters, including alkaline phosphatase (ALP), alanine aminotransferase (ALT), aspartate aminotransferase (AST), albumin (ALB), creatinine (CREA), uric acid (UA) and urea nitrogen (BUN), are analyzed and compared among these groups. The results show that the phototherapy has a lower effect on these indices and liver and kidney functions (Fig. S20†). Moreover, the blood routine examination reveals that the common indices of red blood cells (RBCs), white blood cells (WBCs), lymphocytes (Lymph), *etc.* in the Py-NIR NPs + L group are normal based on a reference (Table S1†). In contrast, all these indices are abnormal in other groups such as saline, saline + L, and Py-NIR NPs as a result of the proliferation of the tumor. This indicates that with the aid of Py-NIR NPs, excellent phototherapeutic effect has been realized, and such a treatment will not cause a negative effect on the body. Then, all these mice are sacrificed while their main organs, the heart, liver, spleen, lung and kidney, are collected for the H&E staining analysis. The H&E staining images from Py-NIR NPs-related groups show no significant changes in cell morphology when compared with those from the control groups, confirming that no damage or inflammation is caused to the normal tissues under all the treatments (Fig. S21†). All these display a good biocompatibility of Py-NIR NPs for potential application.

## Conclusions

In this work, we propose a pyrazine-based planar electronic acceptor with strong electron affinity which can be further utilized to design a NIR-II fluorescenct dye. By attaching two triphenylamine donors to this acceptor, a NIR-II AIE molecule, namely Py-NIR, is obtained with a basic D–A–D module. The molecule is subsequently fabricated into water-dispersed NPs which show good size, colloidal, photo- and thermal stability, and excellent biocompatibility. Thanks to the planar electronic acceptor, distinct NIR I absorption at ∼670 nm and NIR-II emission at ∼1100 nm can be obtained in Py-NIR NPs. We have proved that the planar electronic acceptor can not only contribute to the optical properties but also balance the molecular excited-state decay paths to facilitate a remarkable photothermal conversion. The special construction of the acceptor enables a strong repulsion between the donor units to provide a flexible molecular conformation, which is crucial in the AIE effect. Moreover, the structural twist between the D–A pair can induce a small Δ*E*_ST_ of 0.14 eV to boost the ISC for triplet sensitization. Such behavior can be further tuned using the planarity of the introduced acceptor, while a type I mechanism is dominant to realize an obvious OH˙ formation. Therefore, the synergetic properties of NIR-II fluorescence, photothermal conversion, and type I photosensitization have been obtained with the aid of the planar electronic acceptor. In addition, the combined *in vivo* imaging and therapeutic experiments are evaluated based on orthotopic 4T1 breast tumor bearing mice. With the Py-NIR NPs, the fluorescence imaging in the NIR-II window shows a good target ability, high sensitivity and contrast towards tumors. Meanwhile, as a result of the thermal effect, PTI and PAI can be performed simultaneously which have compensatory functions for fluorescence imaging. Moreover, the good phototherapeutic properties of Py-NIR NPs make them very qualified for tumor therapy. Our work has provided a facile way to obtain probes for combined imaging and therapeutic applications with planar electronic acceptors and created a new opportunity for developing NIR-II fluorescent dyes.

## Data availability

The datasets supporting this article have been uploaded as part of the ESI.†

## Author contributions

M. C., D. W., and B. Z. T. conceived and designed the experiment. M. C. and M. X. synthesized and characterized the materials. Z. Z., M. K., and X. L. carried out the biological experiment. R. L. conducted the spectral analysis. J. L. conducted the theoretical calculation. X. He carried out the TEM test. C. Z. evaluated the stability of nanoparticles. M. C. wrote the paper. H. F., J. W. Y. L., D. W., and B. Z. T. provided suggestions in the study and manuscript. All authors approved the final version of the manuscript.

## Conflicts of interest

There are no conflicts to declare.

## Supplementary Material

SC-015-D3SC06886B-s001
