# Supplementary material for: A planar electronic acceptor motif contributing to NIR-II AIEgen with combined imaging and therapeutic applications
Source: Chem Sci. 2024 Apr 1;15(18):6777–88. doi: 10.1039/d3sc06886b (PMC11077540; doi:10.1039/d3sc06886b)
Supplement: SC-015-D3SC06886B-s001 [file SC-015-D3SC06886B-s001.pdf]

Electronic Supplementary Material (ESI) for Chemical Science.
This journal is © The Royal Society of Chemistry 2024

Supporting Information

A Planar Electronic Acceptor Motif Contributing to NIR-II AIEgen with Combined Imaging and Therapeutic Applications

Ming Chen,‡*[a] Zhijun Zhang,‡[b] Runfeng Lin,[a] Junkai Liu,[c] Meizhu Xie,[a] Xiang He,[a] Canze Zheng,[a] Miaomiao Kang,[b] Xue Li,[b] Hai-Tao Feng,[e] Jacky W. Y. Lam,[c] Dong Wang,*[b] Ben Zhong Tang*[c,d]

[a] College of Chemistry and Materials Science, Jinan University, Guangzhou 510632, China. E-mail: chenming@jnu.edu.cn

[b] Center for AIR Research, College of Materials and Engineering, Shenzhen University, Shenzhen 518060, China. E-mail: wangd@szu.edu.cn

[c] Department of Chemistry, Hong Kong Branch of Chinese National Engineering Research Center for Tissue Restoration and Reconstruction, Guangdong-Hong Kong-MacauJoint Laboratory of Optoelectronic and Magnetic Materials, The Hong Kong University of Scienceand Technology, Clear Water Bay, Kowloon, Hong Kong 999077, China

[d] School of Science and Engineering, Shenzhen Institute of Aggregate Science and Technology, The Chinese University of Hong Kong, Shenzhen (CUHK-SZ), Guangdong, China. E-mail: tangbenz@cuhk.edu.cn

[e] AIE Research Center, Shaanxi Key Laboratory of Photochemistry, College of Chemistry and Chemical Engineering, Baoji University of Arts and Sciences, Baoji 721013, China

‡ These authors contributed equally to this work.

Experimental Section

Materials and Instrumentation

The chemicals including 3,6-Dibromo-9,10-phenanthrenequinone (**1**) and 5,6-diaminopyrazine-2,3-dicarbonitrile (**4**) were purchased from Energy Chemical and TCI, etc., and used directly without further purification. Tetrahydrofuran (THF) was distilled from sodium/benzophenone under nitrogen before use. ^{1}H and ^{13}C spectra were recorded on a Bruker AVIII 400 MHz NMR spectrometer using $CDCl_3$ or CD_2Cl_2 as solvent. High resolution mass spectra (HRMS) were measured on a Finnigan MAT TSQ 7000 mass spectrometer operating in MALDI-TOF mode. UV-Vis spectra were measured on a SHIMADZU UV-2600i UV-Vis spectrophotometer. Fluorescence spectra were performed on EDINBURGH FS5/FLS 1000 spectrofluorometers. TEM image was taken with a JEM-1400Flash JEOL transmission electron microscope. DLS was performed with a NANO ZS (Malvern Panalytical Co., UK) at a fixed angle of 90°. The cellular fluorescence images were taken with a confocal laser scanning microscope (CLSM, ZEISS-LSM900). The cell viability was investigated by CCK-8 kit, and the absorbance of the samples were recorded at 450 nm with a microplate reader (synergyH1, BioTek). The cell apoptosis was investigated with a BD FACSAria SORP fluorescence activated cell sorting (FACS). Temperature change and photothermal images were studied by an E6 camera (FLIR System). The *in vivo* fluorescence imaging was performed with a commercial NIR-II *in vivo* imaging system (MARS-HS, Artemis Intelligent Imaging). Photoacoustic imaging was investigated based on a commercial optoacoustic imaging system (Vevo LAZR, Fuji Film Visual Sonics). Phototherapeutic experiments were conducted by an 808 nm infrared semiconductor laser (Changchun radium photoelectric technology).

Synthesis of 3,6-bis(4-(diphenylamino)phenyl)phenanthrene-9,10-dione (3)

Into a 250 mL round bottom flask was added 3,6-dibromo-9,10-phenanthrenequinone (**1**, 1.0 g, 2.73 mmol), 4-(diphenylamino)phenylboronic acid (**2**, 1.7 g, 5.88 mmol), $Pd(PPh_3)_4$ (300 mg, 0.26 mol), 50 mL of THF and 25 mL of K_2CO_3 aqueous solution (2M) under nitrogen. The mixture was allowed for reflux at 80 °C overnight. After that, the mixture was cooled down to the room temperature and extracted by dichloromethane. The collected organic phase was washed by water several times, dried under anhydrous sodium sulfate and condensed by reduced pressure. The crude product was purified on a silica geo column with petroleum ether/dichloromethane (v/v = 1:2) as eluent. A purple powder of 1.76 g was obtained in a yield of 92.7 %. ^{1}H NMR (400 MHz, $CDCl_3$),

8.26–8.23 (m, 4H), 7.66–6.64 (m, 2H), 7.60–7.58 (d, 4H), 7.33–7.29 (t, 8H), 7.19-7.16 (m, 12H), 7.12–7.08 (t, 4H). δ (ppm): ^{13}C NMR (100 MHz, $CDCl_3$), δ (ppm): 180.0, 149.0, 148.1, 147.2, 136.2, 132.2, 131.2, 129.5, 129.4, 128.1, 127.4, 125.1, 123.8, 122.8, 121.7.

Synthesis of Py-NIR

Into a 100 mL round bottom flask was added **3** (500 mg, 0.72 mmol), 5,6-diaminopyrazine-2,3-dicarbonitrile (**4**, 150 mg, 0.94 mmol), 20 mL of 1,2,4-trimethylbenzene and trace *p*-toluenesulfonic acid. The mixture was allowed for stir at 150 °C for 4 days. After that, the mixture was condensed by reduce pressure and the crude product was purified on a silica geo column with petroleum ether/dichloromethane (v/v = 1:1) as eluent. A black powder of 70 mg was obtained in a yield of 11.9 %. ^{1}H NMR (400 MHz, CD_2Cl_2), δ (ppm): 8.43–8.41 (d, 2H), 7.71 (s, 2H), 7.41–7.37 (t, 8H), 7.29–7.24 (m, 14H), 7.22–7.19 (t, 4H), 7.09–7.07 (d, 4H). ^{13}C NMR (100 MHz, CD_2Cl_2), δ (ppm): 149.2, 148.8, 146.8, 144.2, 143.1, 132.7, 131.9, 129.8, 129.6, 1248.5, 127.6, 126.1, 125.7, 125.4, 124.3, 121.5, 119.0, 113.1. HRMS (MALDI-TOF): *m/z* 818.2910 ($[M]^+$), calcd for $C_{56}H_{34}N_8$ 818.2906.

Theoretical calculation

The optimization of S_1 conformation and calculation of electrostatic potential map were carried out with a Gaussian 09 package[1] at TD-PBE0/6-31G(d,p). Empirical dispersion was taken to obtain a better description of the weak interaction. The SMD model was adopted to simulate the THF environment. The electron-vibration coupling was investigated in MOMAP 2022B.[2-4]

Fabrication of NPs

Py-NIR (1 mg) and DSPE-mPEG$_{2000}$ (10 mg) were first dissolved in 1 mL of THF, which was then added into 10 mL of water dropwise in 1 min under ultrasound. Followed by another 2-minute ultrasound, the organic solvent was removed by dialysis (3500 kDa molecular weight cutoff) and the NPs were concentrated by centrifuge ultrafiltration. The concentration of NPs based on Py-NIR was calculated based on a standard curve by the UV-Vis spectra analysis.

Photothermal conversion evaluation

To evaluate the effect of Py-NIR NPs concentration on the photothermal conversion properties, the NPs with different concentration of 25, 50, 75, 100 and 150 μM (based on dye) were irradiated by 808 nm laser (1.0 W cm^{-2}). Similarly, to evaluate the powder dependence, the 808 nm laser with different laser power densities (0.6, 0.8, 1.0, 1.2, 1.4 W cm^{-2}) were applied to the photothermal experiments. The photothermal stability was studied by a heating-cooling experiment with 6 cycles.

The variation in temperature was recorded by a FLIR E6 camera. The photothermal conversion efficiency of NPs was evaluated by the reported method.[5]

ROS evaluation

The indicators of DCFH-DA, ABDA, DHR123 and HPF were employed to evaluate the overall ROS, $^1O_2$, $O_2^{\bullet-}$ and $OH^{\bullet}$, respectively. 3 mL of PBS containing activated DCFH-DA (5 μM), ABDA (40 μM), DHR123 (5 μM) or HPF (5 μM), and Py-NIR NPs (1 μM, based on dye) were irradiated by the 808 nm laser (1.0 W cm^{-2}) for different time intervals. The fluorescence spectra of DCFH-DA, DHR123 and HPF were obtained with excitations of 488 nm, 495 nm and 490 nm, respectively, while the emission intensities at 524 nm, 524 nm and 515 nm were recorded. The UV-Vis spectra of ABDA were obtained with their absorption intensities at 378 nm recorded.

Cell culture

Mouse breast cancer 4T1 cell line was provided by Chinese Academy of Science Cell Bank for Type Culture Collection, and grown in 1640 culture medium having 10% FBS and 1% antibiotics (penicillin-streptomycin) at 37 °C under 5% CO_2.

Intracellular ROSs generation

4T1 cells were seeded in glass bottom dish and cultured for 24 h, After that, the cells were incubated with or PBS or Py-NIR NPs (50 μM, based on dye) in fresh medium for 12 h, followed by incubated with DCFH-DA (10 μM) in 1 mL fresh serum-free medium at 37 °C for another 20 min. The cells were washed and irradiated by 808 nm laser (1.0 W cm^{-2}) for 5 min if necessary and imaged by CLSM. Excitation wavelength: 488 nm; emission filter: 500–550 nm.

Live-dead cell staining

4T1 cells were seeded in glass bottom dish and cultured for 24 h. Then, the cells were divided into four groups with treatments of PBS, PBS + laser, Py-NIR NPs, and Py-NIR NPs + laser, respectively. For all groups, the cells were incubated for 12 h before further treatments. For groups involving light irradiation, the 808 nm laser (1.0 W cm^{-2}) was used to irradiate the cells for 5 min. The concentration of Py-NIR NPs was 50 μM based on dye. After these treatments, the cells were incubated at 37 °C for another 30 min, and then stained by PI (60 μg mL^{-1}) and FDA (100 μg mL^{-1}) for 10 min. After that, the cells were gently washed and then imaged by CLSM. Excitation wavelength: 488 nm (FDA) and 561 nm (PI); emission filter: 500–550 nm (FDA) and 600–700 nm (PI).

Cell apoptosis evaluation

4T1 cells (10^5 cells per well) were seeded and cultured in 6-well plates for 24 h. After that, the cells were incubated with the Py-NIR NPs (50 μM, based on dye) in the culture media for 12 h and irradiated by 808 nm laser (1.0 W cm^{-2}) for 5 min. The cells treated with PBS, Py-NIR NPs and PBS plus laser were set as the controls. Then, the cells were incubated at 4 °C for another 0.5 h, washed with PBS, and collected by centrifugation at 1000 rpm for 5 min at 4 °C. Afterwards, the cells were stained with an Annexin V-FITC/PI Apoptosis Detection Kit based on manufacturer's instructions and analyzed by FACS.

Phototoxicity test

4T1 cells were seeded on a 96-well plates at a density of 5×10^3 cells per well and cultured for 24 h. After removing the culture medium, the new mediums containing Py-NIR NPs with different concentrations (0, 10, 20, 30, 40 and 50 μM, based on dye) were adopted. After 12 h incubation, the cells were irradiated by 808 nm laser (1.0 W cm^{-2}) for 5 min. At the same time, the cells incubated with the NPs in the absence of light irradiation were taken for the dark cytotoxicity study. After further 12 h incubation, the culture media were removed, and the cells were washed with PBS for three times and incubated with fresh FBS-free medium containing 10% CCK-8 for 2 h in the dark. Then, the absorption wavelength at 450 nm of the products was recorded with a microplate reader. The results were embodied as the viable percentage of cells after light irradiation versus the cells without light irradiation. The relative cell viability was calculated based on the following formula: Cell viability (%) = $(OD_{sample} - OD_{background})/(OD_{control} - OD_{background}) \times 100\%$.

Animals and tumor model

All the animal experiments conducted in this work were approved by the Administrative Committee on Animal Research in Shenzhen Graduate School, Peking University (SYXK(YUE)2017-0172). Female BALB/c mice aged 4~5 weeks were provided by Beijing Vital River Laboratory Animal Technology. The mice were housed under a pathogen-free condition and fed with standard laboratory water and chow. Orthotopic 4T1 breast tumor model was established by orthotopical injection of 4T1 cells (5×10^5) suspended in PBS into the upper right mammary fat pads. The 4T1 tumor bearing mice were subsequently employed when the tumor volumes reached ~ 100 mm^3.

In vivo NIR-II fluorescence, photoacoustic and photothermal imaging

For NIR-II fluorescence or photoacoustic imaging, the orthotopic 4T1 breast tumor bearing mice were administered with 200 μL of Py-NIR NPs (2 mM, based on the dye) via tail vein. Then, the in vivo NIR-II fluorescence images were captured at predetermined time intervals (3, 6, 12 and 24 h) of post-injectionon by a NIR-II in vivo imaging system with the long pass (LP) filter of 1000 nm under an excitation wavelength of 808 nm. Besides, the in vivo photoacoustic imaging was collected on a commercial optacoustic imaging system at the same time intervals after intravenous injection of the NPs. The NIR-II fluorescence and photoacoustic images at 0 h were obtained before the NPs injection. In order to evaluate the distributions of Py-NIR NPs in the tissues, the mice were sacrificed at 24 h post-injection. The major organs including heart, liver, spleen, lung, and kidney, and tumor were excised. Then, they are washed with saline several times followed by NIR-II fluorescence imaging and quantitative analyses. For in vivo photothermal imaging, the infrared thermal images of mice were taken with an IR camera under the irradiation of 808 nm laser (1.0 W cm^{-2}) while they are intravenously injected with 200 μL of Py-NIR NPs (2 mM, based on dye) for 12 h. Meanwhile, the mice treated with saline under the same irradiation condition was employed as a control.

In vivo antitumor efficacy

To evaluate the in vivo antitumor efficacy of Py-NIR NPs, the orthotopic 4T1 breast tumor bearing mice with the tumor volume reached ~ 100 mm^3 were randomly divided into 4 groups (n = 5), which were Saline, Saline + L, Py-NIR NPs and Py-NIR NPs + L, respectively. For these groups, 200 μL of saline or Py-NIR NPs (2 mM, based on dye) were intravenously injected into the mice before further treatments. In groups needing light irradiation, the 808 nm laser (1.0 W cm^{-2}) was employed to irradiate the tumor site in each group for 10 min after intravenous injection of saline or Py-NIR NPs (2 mM, based on dye) for 12 h. After different treatments, the mouse body weight and tumor volume were recorded every 3 days during the 15-day study duration. The tumor size was measured by a vernier caliper, and the tumor volume (V) was estimated using the formula: $V = (\text{length} \times \text{width}^2)/2$. Relative tumor volume (RTV) was evaluated as $RTV = V/V_0$, while V_0 was the initial tumor volume. Relative body weight (RBW) was evaluated as $RBW = (W-W_0)/W_0$, while W_0 was the initial mouse body weight.

Histological and hematological analyses

At the 15th day of various treatments, the blood samples of the mice were extracted for serum biochemistry and hematology analyses. After that, the mice were sacrificed. The tumors and major

organs including heart, liver, spleen, lung and kidney were excised, fixed in 4% (v/v) formalin saline overnight, embedded in paraffin and then sectioned at the thickness of 5 μm. For the group treated with Py-NIR NPs plus laser, tumor samples were collected at 12 h after light irradiation. Then, the obtained tumor sections from four groups were subjected to H&E, CD31, Ki67 and TUNEL staining and investigated by an inverted optical microscopy to evaluate the histopathological behavior. Besides, the slices of major organs were conducted by H&E staining to evaluate the biosafety.

Statistical Analysis

Data are donated as the mean ± standard deviation (SD). The significance between experimental and control groups was determined by unpaired 2-tailed Student's *t*-test with the GraphPad Prism 7. A value of $p < 0.05$ was considered statistically significant. *$p < 0.05$, **$p < 0.01$, ***$p < 0.001$, ****$p < 0.0001$.

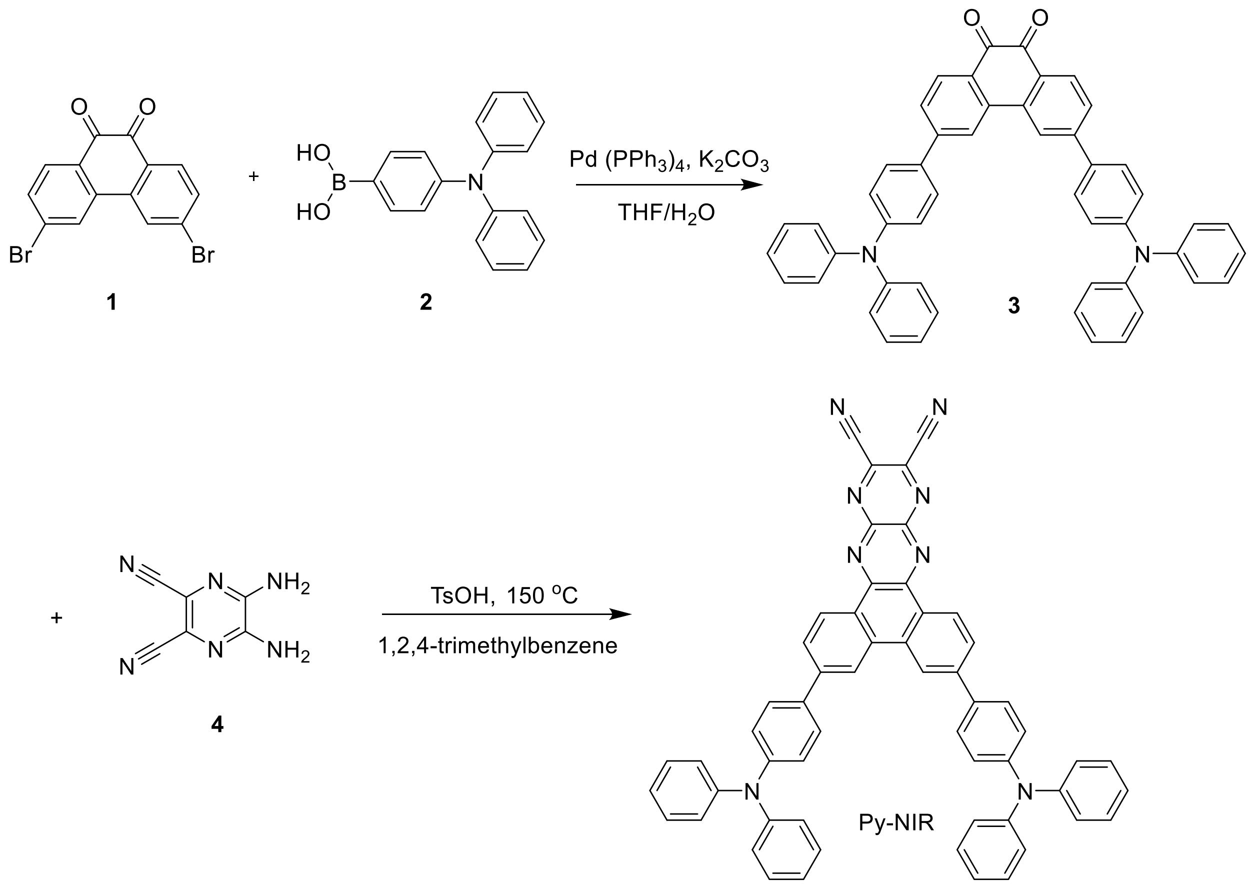

Scheme S1. Synthetic route to Py-NIR.

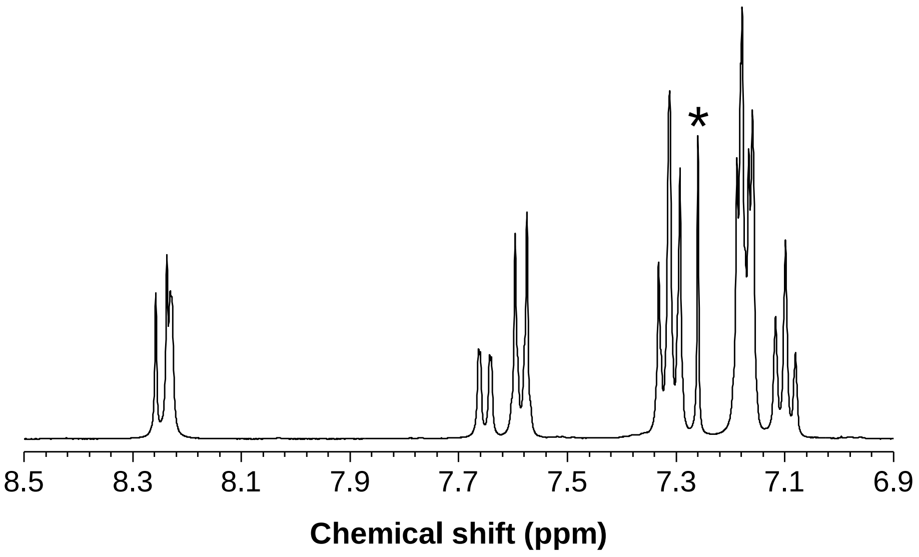

Figure S1. ^{1}H NMR spectrum of **3** in $CDCl_3$. The solvent is marked with asterisk.

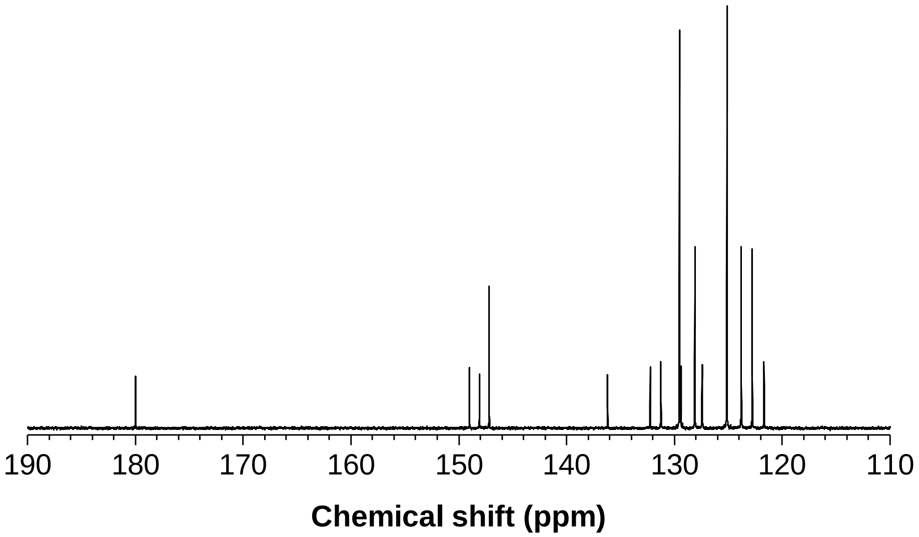

Figure S2. ^{1}H NMR spectrum of **3** in $CDCl_3$.

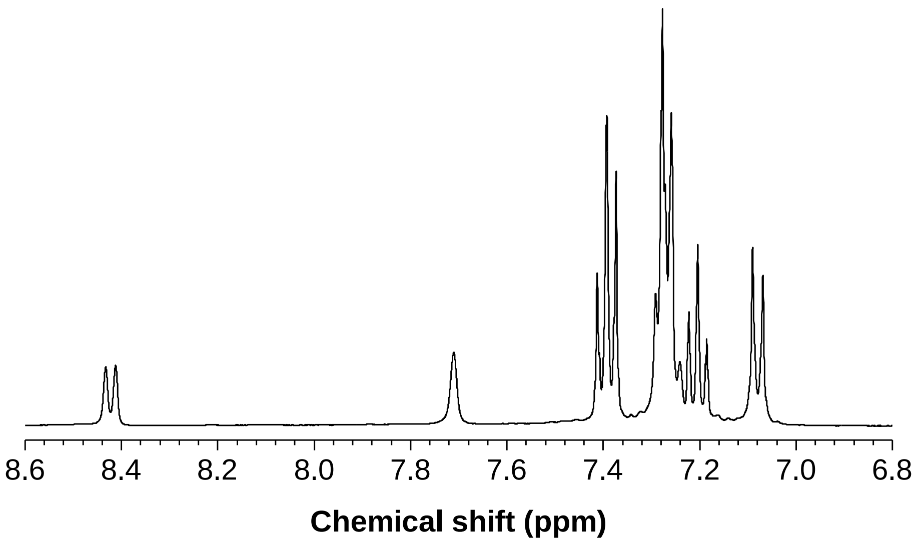

Figure S3. ^{1}H NMR spectrum of Py-NIR in CD_2Cl_2.

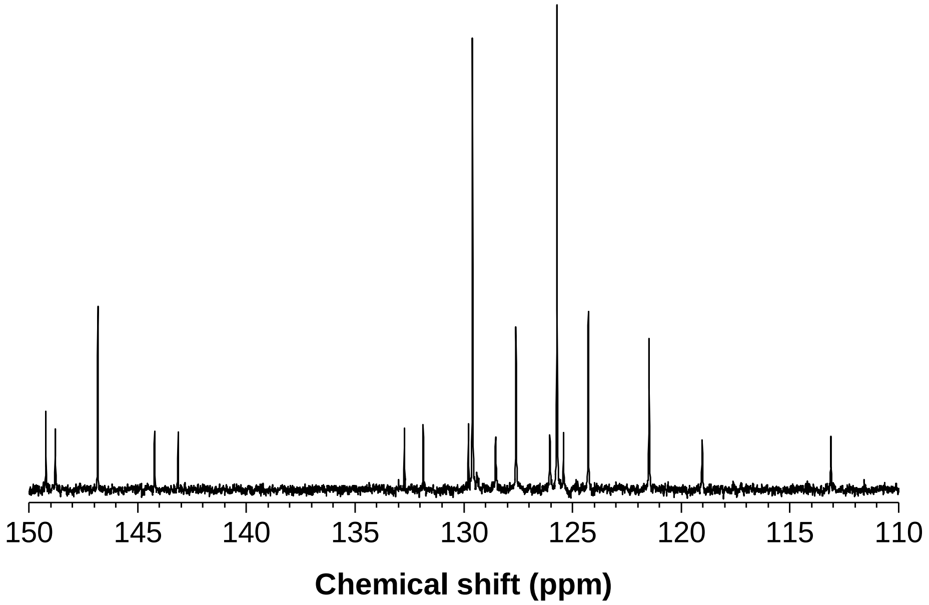

Figure S4. ^{13}C NMR spectrum of Py-NIR in CD_2Cl_2.

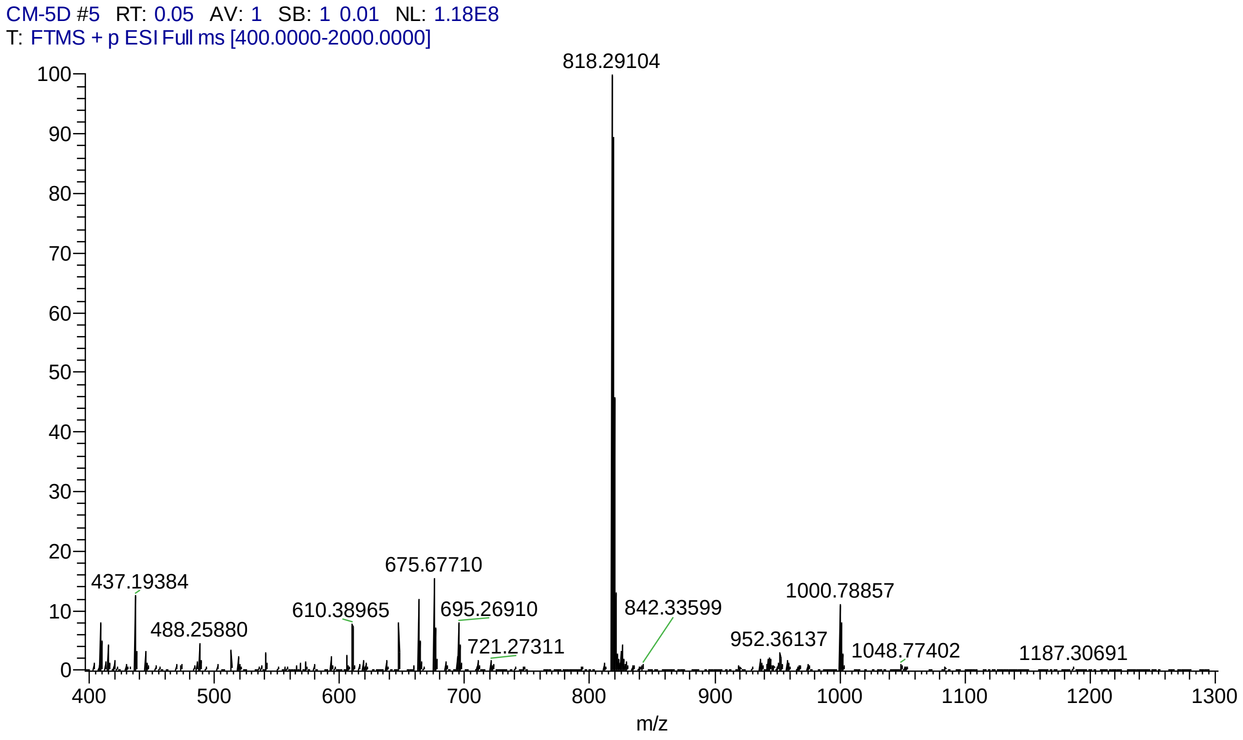

Figure S5. HRMS of Py-NIR.

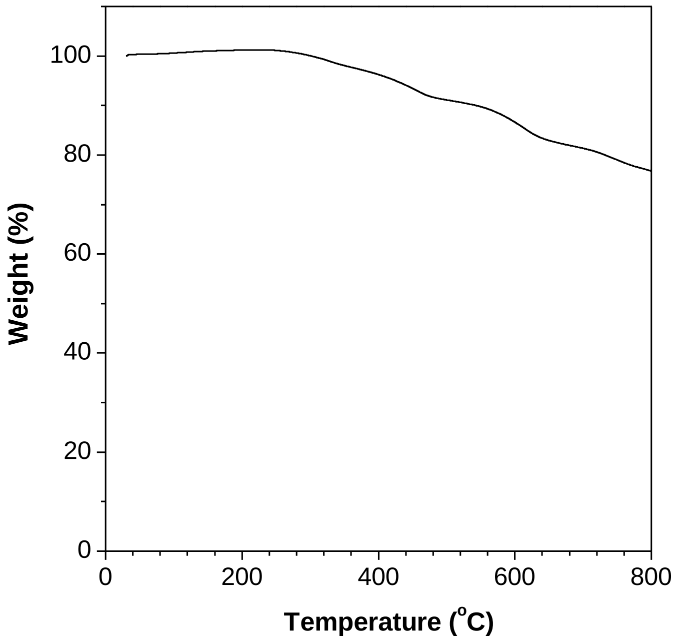

Figure S6. TGA curve of Py-NIR under nitrogen at a heating rate of 10 °C /min.

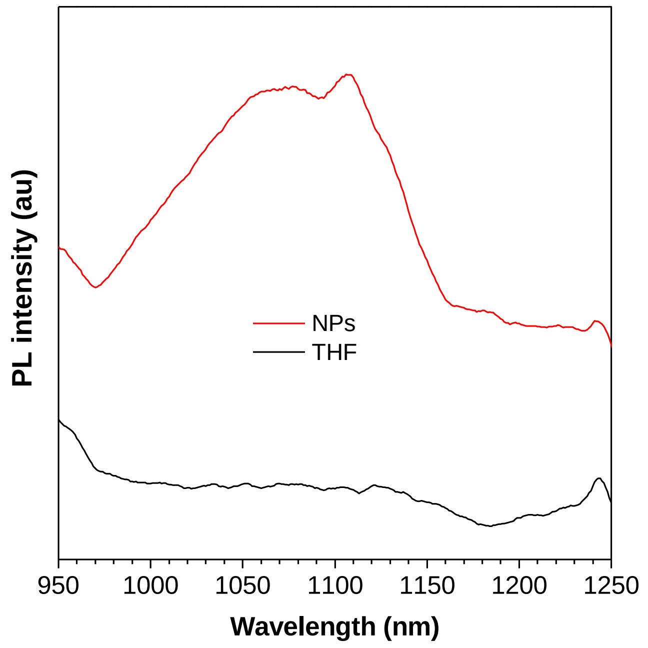

Figure S7. Fluorescence spectra of Py-NIR in NPs and THF. Excitation wavelength = 660 nm.

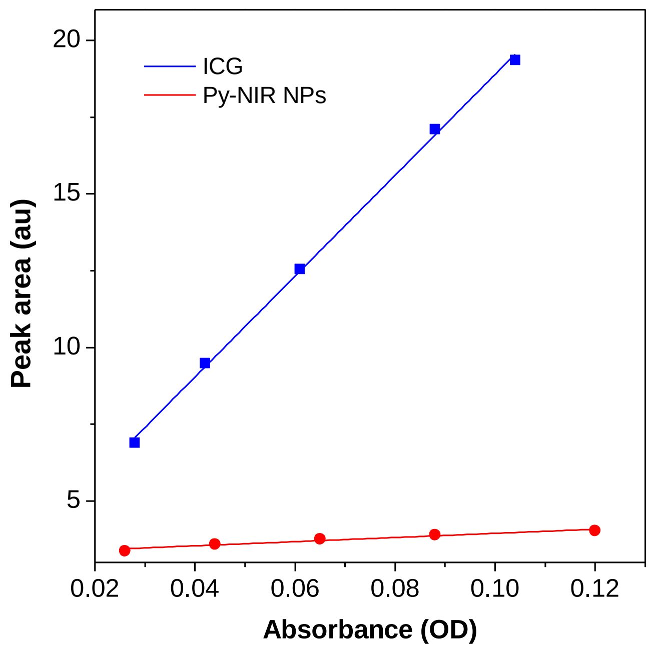

Figure S8. Deducing the quantum yield (QY) of Py-NIR NPs from the linear fitting curves of emission peak integrate areas versus absorbance by using ICG as a standard.[6,7] QY(Py-NIR NPs) = QY(ICG) × slope(Py-NIR NPs) / slope(ICG) = 0.07%.

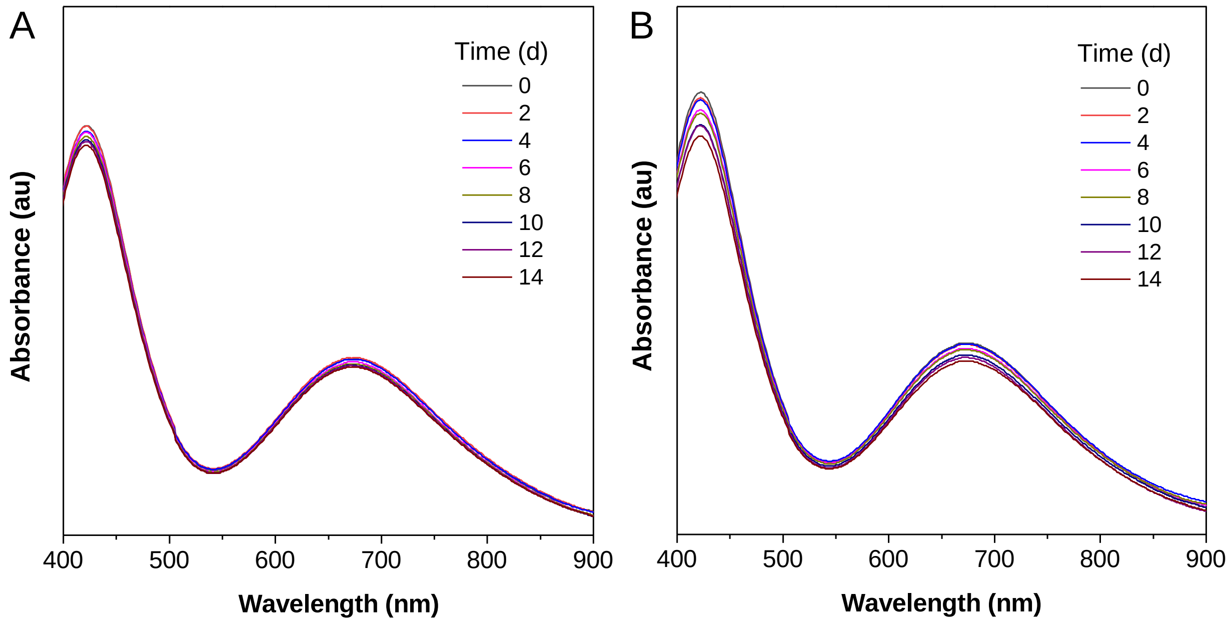

Figure S9. UV-vis spectra of Py-NIR NPs in (A) water and (B) PBS during two weeks.

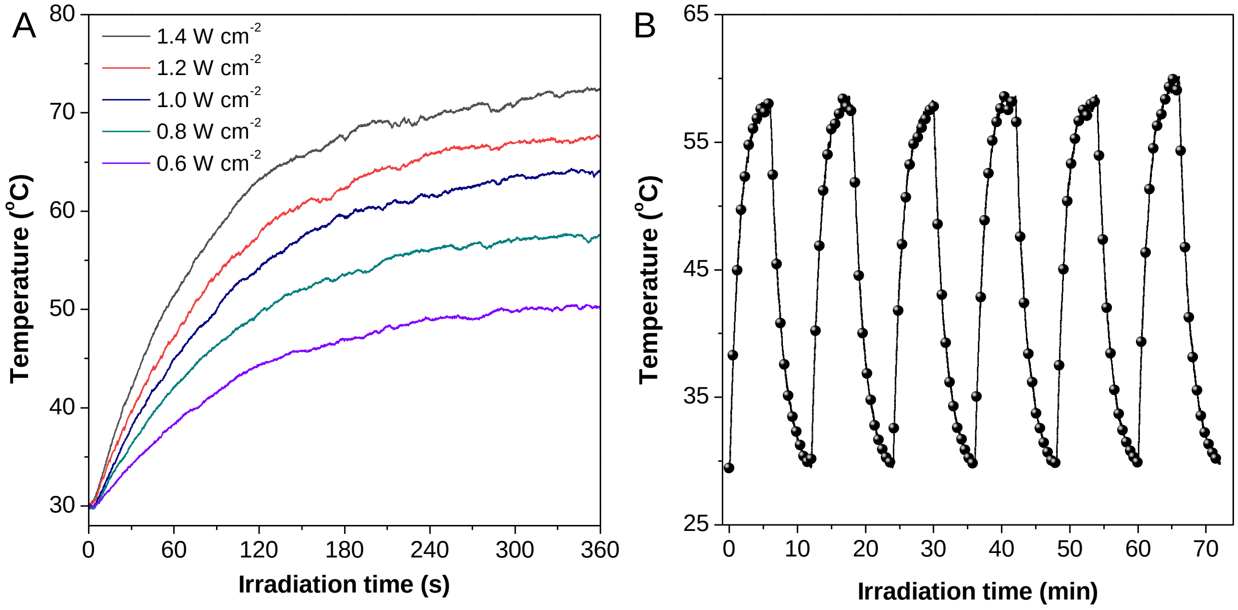

Figure S10. (A) Photothermal curves of Py-NIR NPs under different 808 nm laser powder densities. (B) Heating-cooling experiment of Py-NIR NPs with 6 cycles.

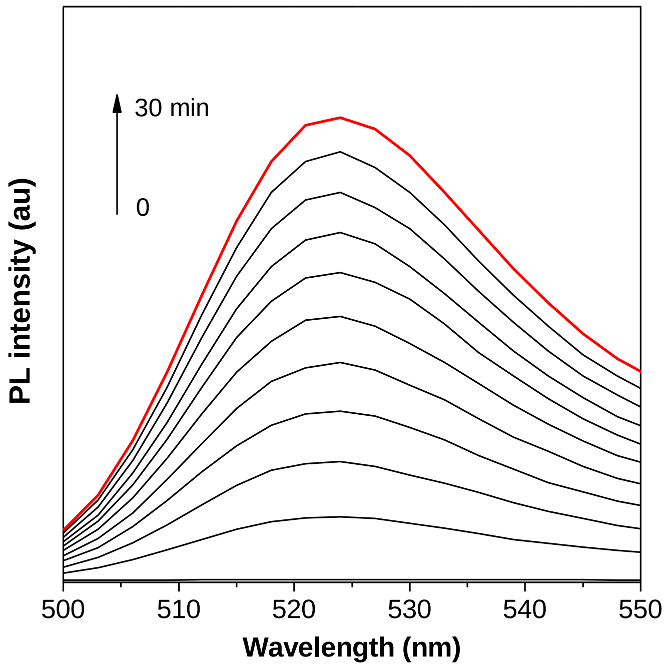

Figure S11. PL spectra of DCFH-DA in the presence of Py-NIR NPs under continuous 30-minute 808 laser irradiation (1 W cm^{-2}).

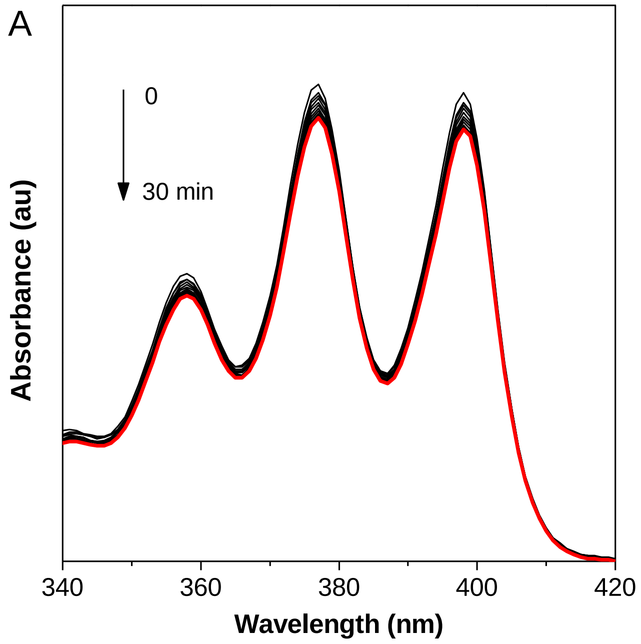

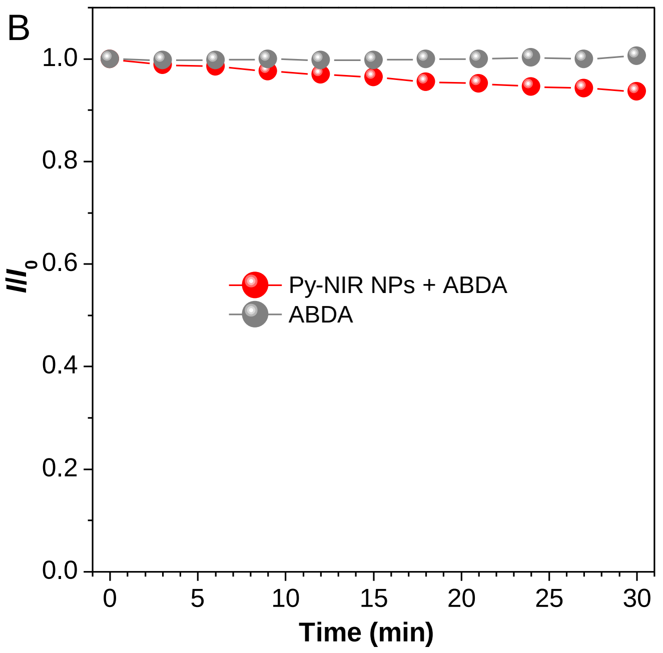

Figure S12. (A) UV-Vis spectra of ABDA in the presence of Py-NIR NPs under continuous 30-minute 808 laser irradiation (1 W cm^{-2}). (B) Changes of relative absorption intensities versus time.

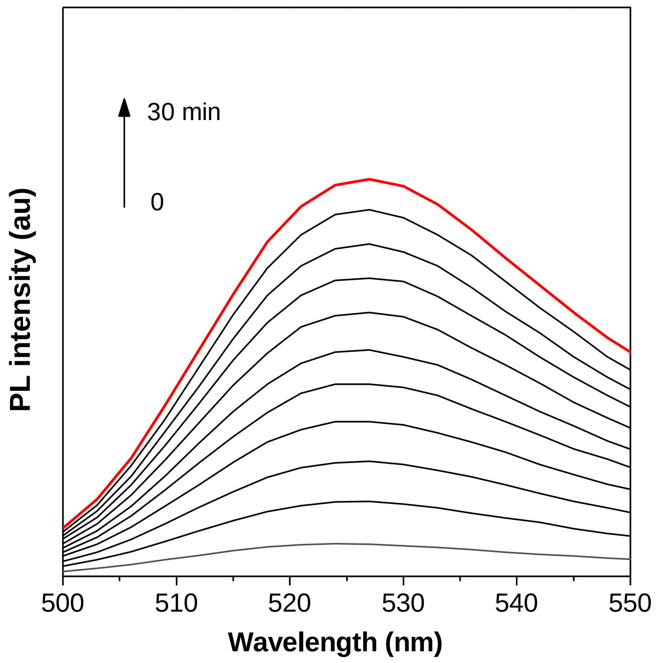

Figure S13. PL spectra of DHR123 in the presence of Py-NIR NPs under continuous 30-minute 808 laser irradiation (1 W cm^{-2}).

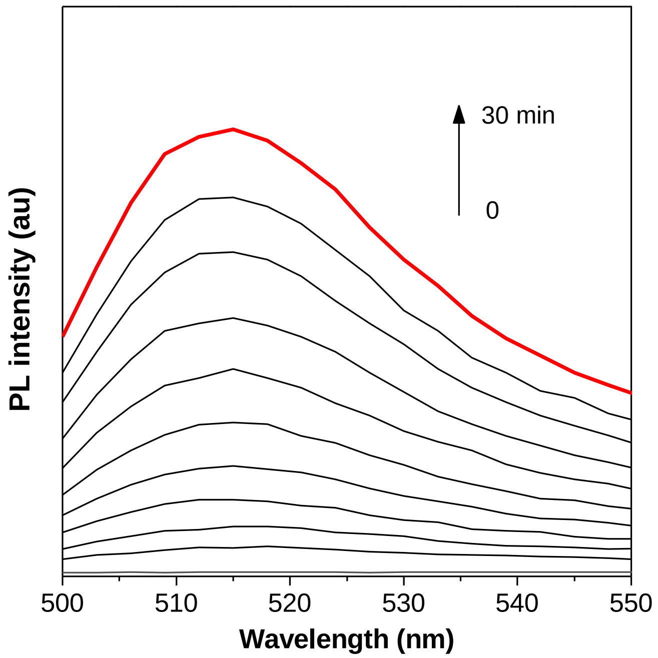

Figure S14. PL spectra of HPF in the presence of Py-NIR NPs under continuous 30-minute 808 laser irradiation (1 W cm^{-2}).

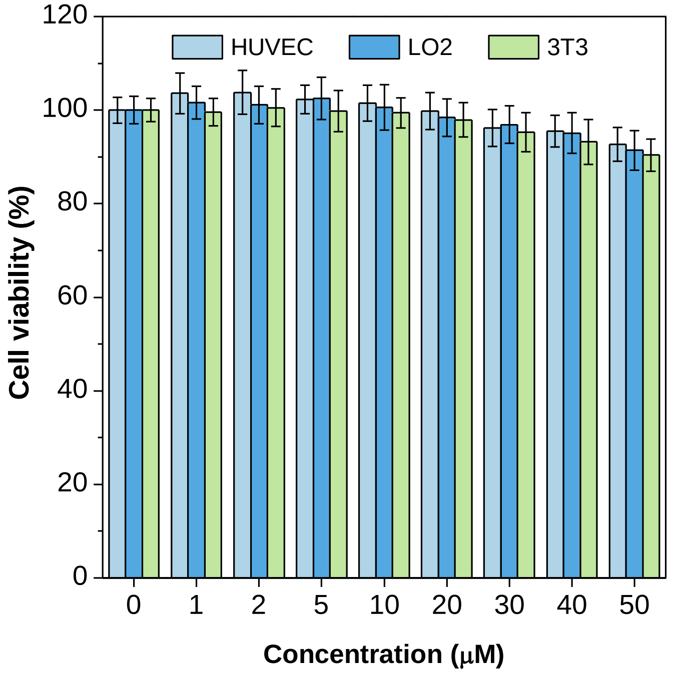

Figure 15. Cell viabilities of HUVEC (human umbilical vein endothelial cell), LO2 (human normal liver cell) and 3T3 (mouse fibroblast cell) after incubation of Py-NIR NPs with different concentrations for 24 h.

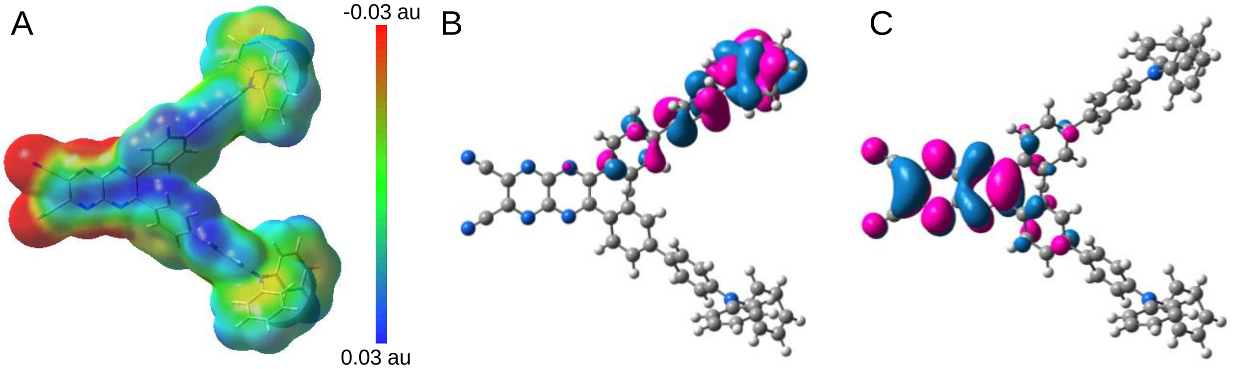

Figure S16. (A) Electrostatic potential map based on optimized S_1 conformation of DCP-PTPA. (B) HOMO and (C) LUMO distributions on S_1 conformation.

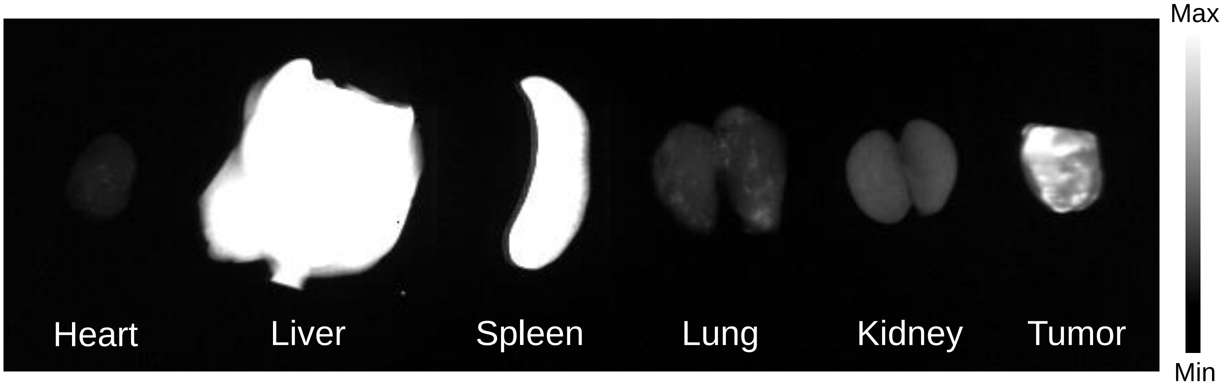

Figure S17. NIR-II fluorescence imaging of heart, liver, spleen, lung, kidney and tumor excised from the mice after 24 h post-injection of the Py-NIR NPs.

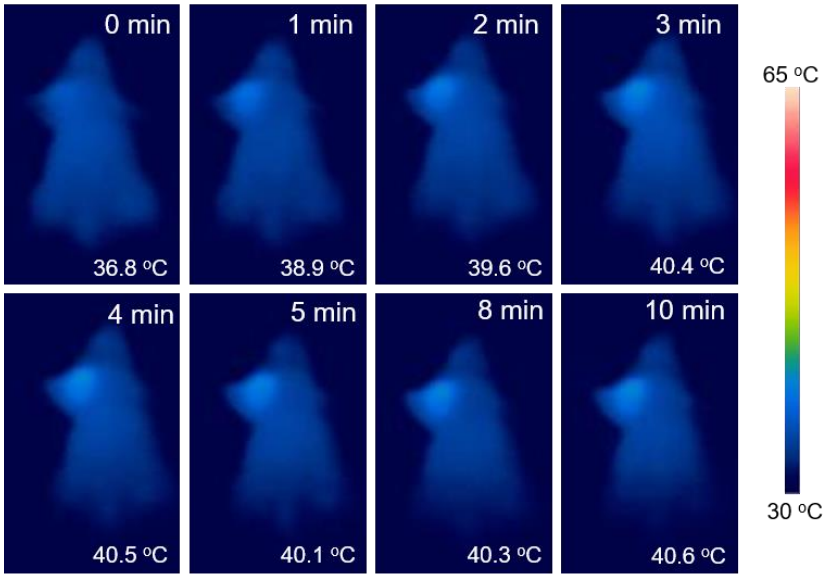

Figure S18. Photothermal images of mice under 808 nm laser excitation (1.0 W cm^{-2}) after 12 h post-injection of the Py-NIR NPs.

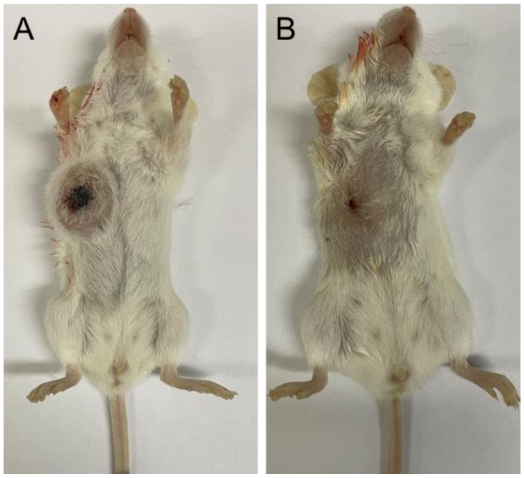

Figure S19. Photograph of 4T1 tumor bearing mice treated with (A) saline and (B) laser plus Py-NIR NPs at day 15.

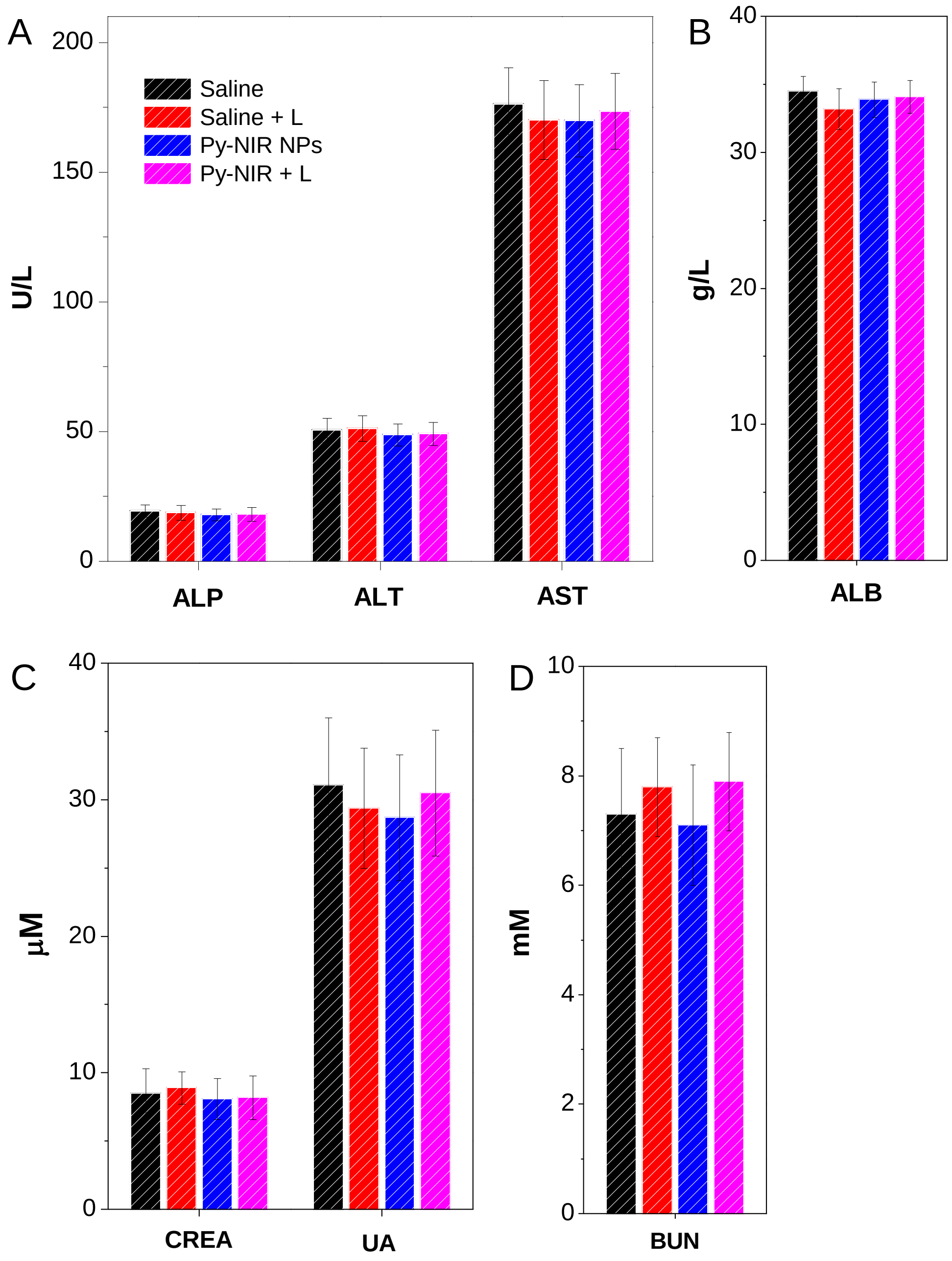

Figure S20. Blood chemistry indices of (A and B) Hapatic function markers and (C and D) renal function markers at day 15. ALP = alkaline phosphatase; ALT = alanine aminotransferase; AST = aspartate aminotransferase; ALB = albumin; CREA = creatinine; UA = uric acid; BUN = urea nitrogen.

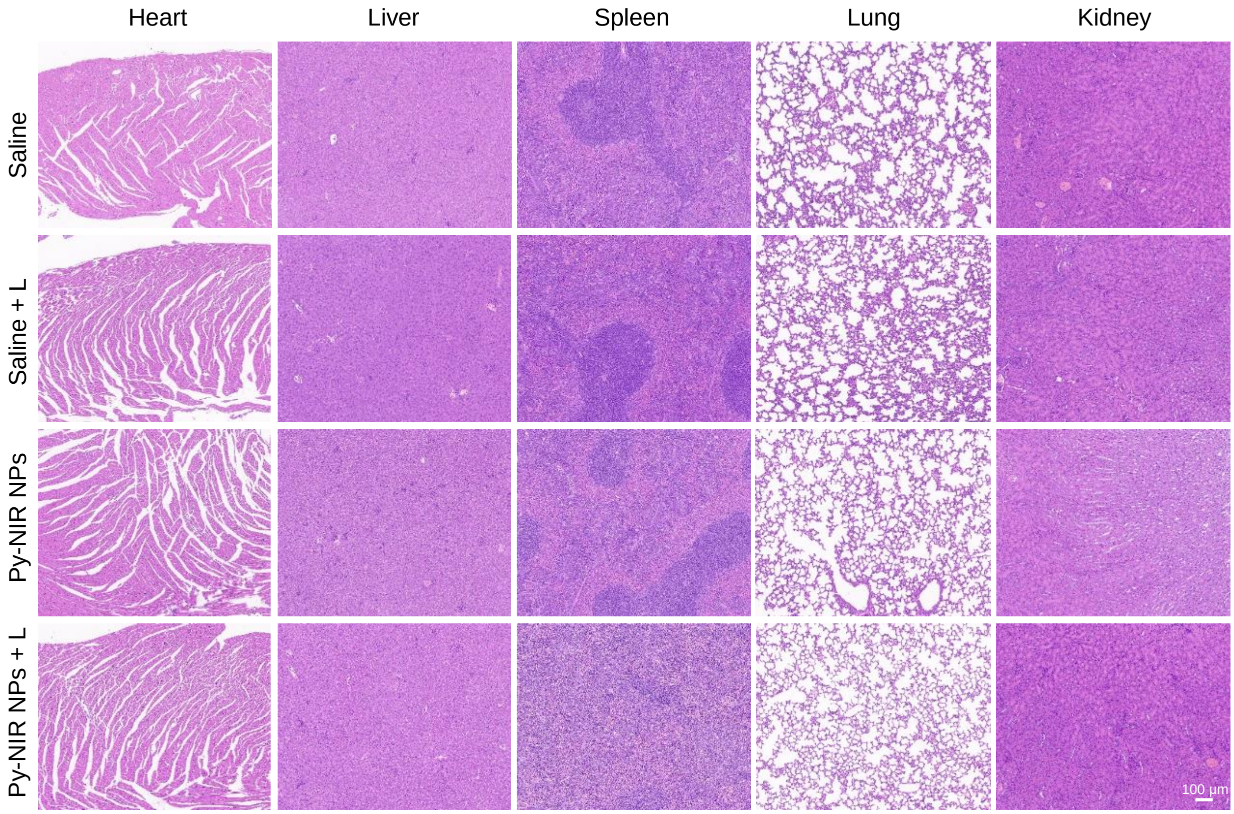

Figure S21. H&E staining of tissue slices of common organs (heart, liver, spleen, lung and kidney) excised from the mice with different treatments at day 15.

Table S1. Routine blood indices of mice after different treatments.

		Saline	Saline + L	Py-NIR NPs	Py-NIR NPs + L	Reference range
WBC	(10^9/L)	164.3 ± 26.2	157.4 ± 30.2	159.9 ± 27.9	4.3 ± 0.7	0.8 ~ 6.8
Lymph	(10^9/L)	21.5 ± 4.7	19.6 ± 4.9	20.3 ± 5.5	4.5 ± 0.8	0.7 ~ 5.7
Mon	(%)	7.9 ± 2.3	8.2 ± 2.5	7.5 ± 2.8	0.2 ± 0.06	0.0 ~ 0.3
Gran	(10^9/L)	149.6 ± 62.6	157.8 ± 60.1	153.2 ± 56.1	1.3 ± 0.6	0.1 ~ 1.8
RBC	(10^{12}/L)	8.2 ± 1.5	8.8 ± 1.9	8.4 ± 1.8	8.1 ± 1.6	6.36 ~ 9.42
HGB	(g/l)	159.4 ± 36.7	160.3 ± 38.2	166.7 ± 39.6	130.8 ± 13.7	110 ~ 143
HCT	(%)	51.6 ± 12.4	50.5 ± 10.8	53.7 ± 14.2	40.1 ± 1.9	34.6 ~ 44.6
MCV	(fl)	48.5 ± 4.2	49.1 ± 3.6	49.9 ± 4.3	52.7 ± 1.7	48.2 ~ 58.3
MCH	(pg)	16.9 ± 2.3	17.1 ± 2.6	17.7 ± 2.9	16.6 ± 1.8	15.8 ~ 19
MCHC	(g/l)	347.1 ± 5.5	348.6 ± 6.1	351.3 ± 5.8	328.8 ± 4.4	302 ~ 353
RDW	(%)	14.2 ± 2.1	14.1 ± 2.5	13.9 ± 2.2	14.3 ± 0.8	13 ~ 17
MPV	(fl)	6.9 ± 1.4	6.8 ± 1.6	6.4 ± 1.8	5.0 ± 0.5	3.8 ~ 6.0

Reference

1. M. J. Frisch, et al. Gaussian 09, Revision D.01, Gaussian, Inc., Wallingford CT, 2016.

2. Z. Shuai, *Chin. J. Chem.* 2020, **38**, 1223–1232.

3. Z. Shuai, Q. Peng, *Phys. Rep.* 2014, **537**, 123–156.

4. Z. Shuai, Q. Peng, *Nat. Sci. Rev.* 2017, **4**, 224–239.

5. Q. Tian, F. Jiang, R. Zou, Q. Liu, Z. Chen, M. Zhu, S. Yang, J. Wang, J. Wang, J. Hu, *ACS Nano* 2011, **5**, 9761–9771.

6. J. Liu, C. Chen, S. Ji, Q. Liu, D. Ding, D. Zhao, B. Liu, *Chem. Sci.*, **2017**, *8*, 2782–2789.

7. Y. Zhang, Y. Jia, S. Zhu, *Smartmat.* **2023**, e1245.
